# Supplementary material for: Effects of Drought and Flooding on Phytohormones and Abscisic Acid Gene Expression in Kiwifruit
Source: Int J Mol Sci. 2023 Apr 20;24(8):7580. doi: 10.3390/ijms24087580 (PMC10143653; doi:10.3390/ijms24087580)
Supplement: Supplementary file 1 [file ijms-24-07580-s001.zip › Water Stress in kiwifruit Supplementary Information v5.pdf]

International Journal of
Molecular Sciences

MDPI

Supplementary materials

The following figures only present phytohormone data that were statistically significant, as shown in Tables S1-S4.

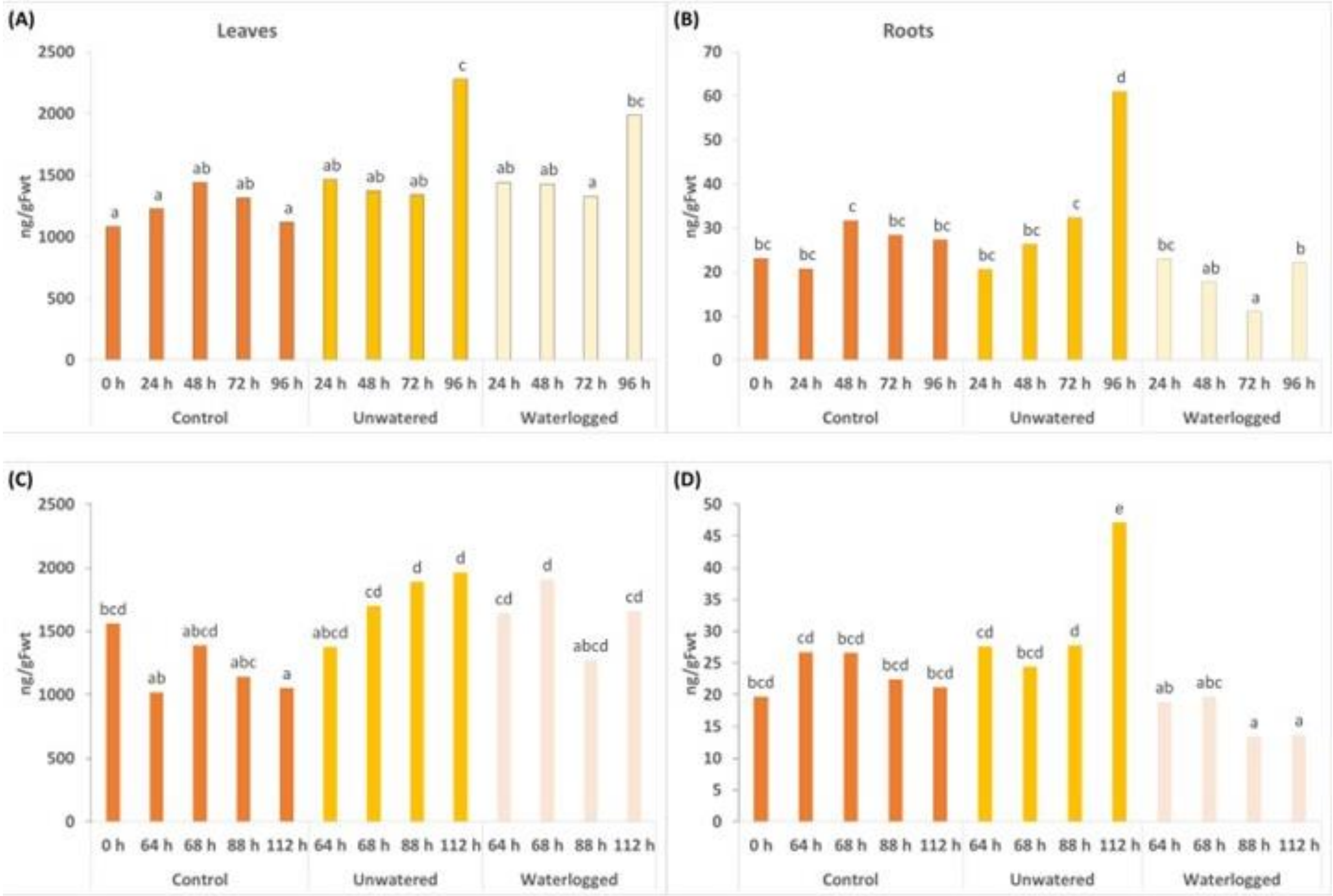

Figure S1. 7-Hydroxy-abscisic acid (7-OH-ABA) concentrations measured in *Actinidia chinensis* var. *chinensis* 'Zesy002' leaves (left panel) and roots (right panel) at various sample times (shown in hours on the x-axes) after application of three treatments in 2020. From time 0 h, plants in the unwatered treatment were not watered and waterlogged plants were subjected to constant water at quarter pot height. Plants in the control treatment received the standard watering regime of 12 minutes, twice daily. There were five replicate plants/time/treatment. Treatments were applied over a 96 h treatment period as shown in **(A)** and **(B)**, and 112 h **(C)** and **(D)**. Different lettering over the bars indicates statistically different differences, as shown by LSD, $p \leq 0.05$, within each experiment and tissue type.

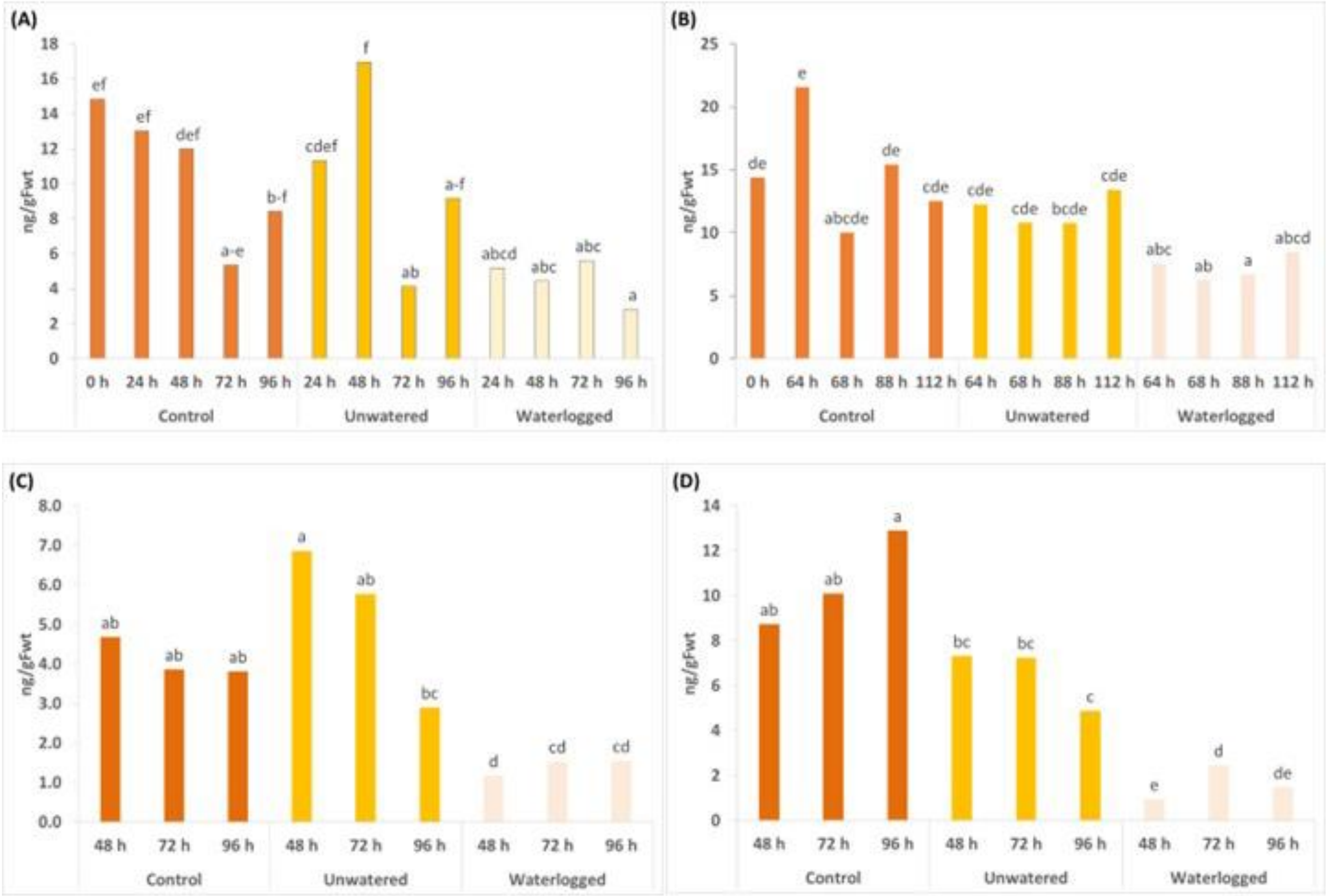

Figure S2. Jasmonic acid (JA) concentrations measured in roots of potted kiwifruit plants at various sample times (shown in hours on the x-axes) after application of three treatments. Starting from time 0 h, plants in the unwatered treatment were not watered and waterlogged plants were subjected to constant water at quarter pot height. Plants in the control treatment received the standard watering regime of 12 minutes, twice daily. There were five replicate plants/time/treatment. Four separate experiments were carried out (two each in 2020 and 2021). Treatments were applied to *Actinidia chinensis* var. *chinensis* 'Zesy002' plants over 96 h **(A)** and over 112 h **(B)** in 2020. An identical experimental setup was used to compare responses between 'Zesy002' **(C)** and *Actinidia chinensis* var. *deliciosa* 'Hayward' **(D)** in 2021. Different lettering over the bars indicates statistically different differences, as shown by LSD, $p \leq 0.05$, within each experiment.

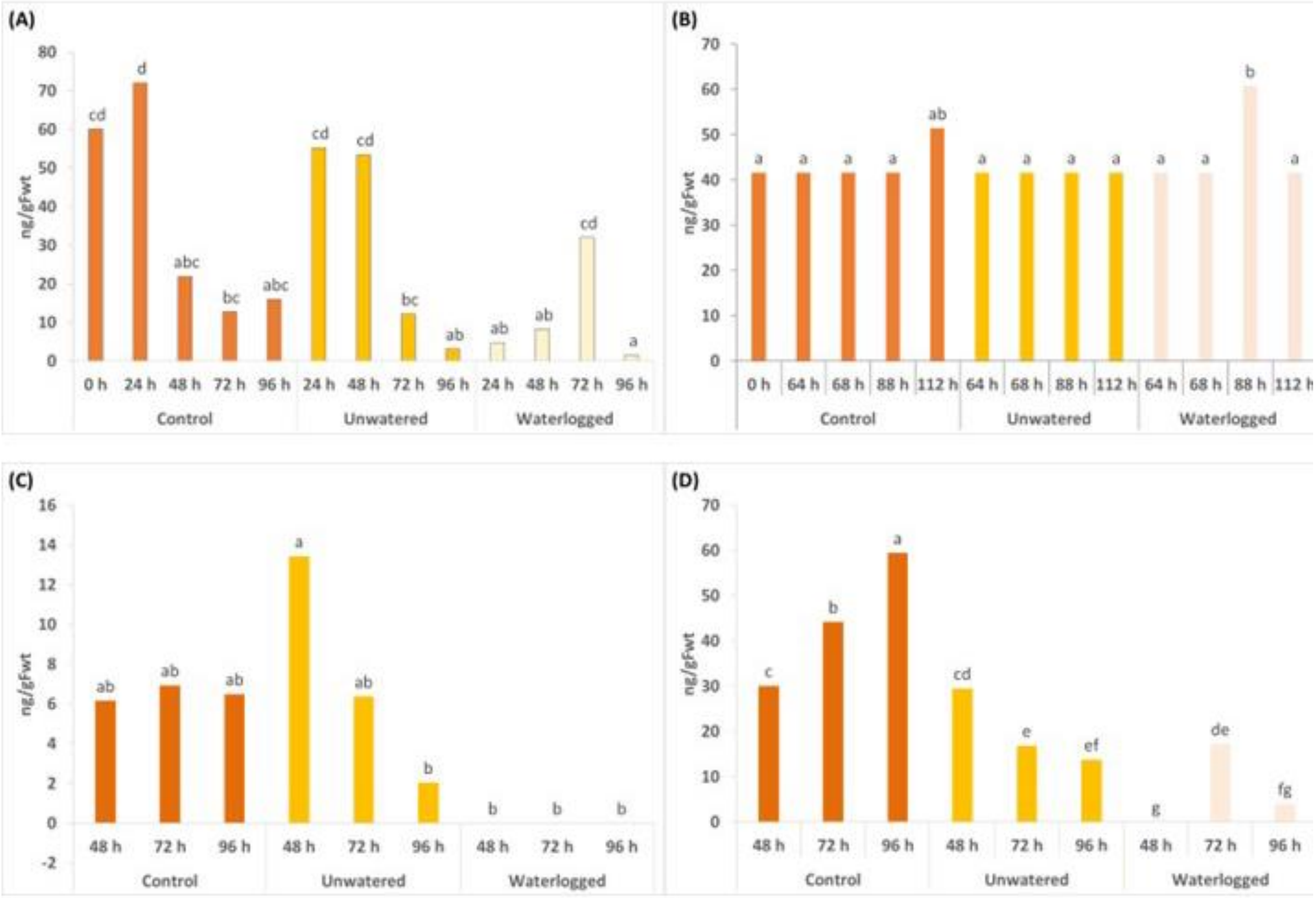

Figure S3. (+/-)-4-(3-Oxo-2-(pent-2-enyl)cyclopentyl) butanoic acid (OPC-4) concentrations measured in roots of potted kiwifruit plants at various sample times (shown in hours on the x-axes) after application of three treatments. Starting from time 0 h, plants in the unwatered treatment were not watered and waterlogged plants were subjected to constant water at quarter pot height. Plants in the control treatment received the standard watering regime of 12 minutes, twice daily. There were five replicate plants/time/treatment. Four separate experiments were carried out (two each in 2020 and 2021). Treatments were applied to *Actinidia chinensis* var. *chinensis* 'Zesy002' plants over 96 h **(A)** and over 112 h **(B)** in 2020. An identical experimental setup was used to compare responses between 'Zesy002' **(C)** and *Actinidia chinensis* var. *deliciosa* 'Hayward' **(D)** in 2021. Different lettering over the bars indicates statistically different differences, as shown by LSD, $p \leq 0.05$, within each experiment.

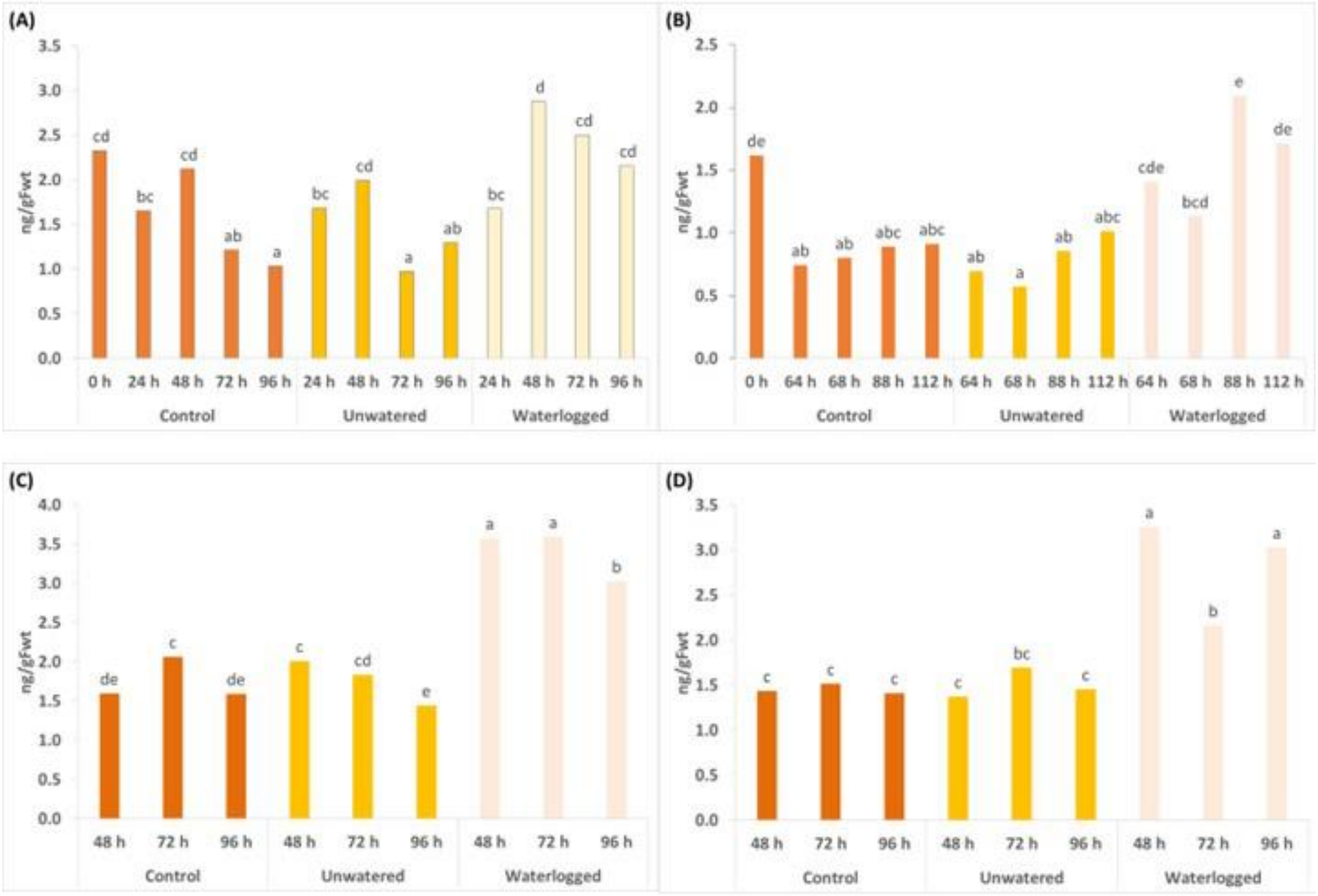

Figure S4. 9,10-Dihydrojasmonic acid (DH-JA) concentrations measured in roots of potted kiwifruit plants at various sample times (shown in hours on the x-axes) after application of three treatments. Starting from time 0 h, plants in the unwatered treatment were not watered and waterlogged plants were subjected to constant water at quarter pot height. Plants in the control treatment received the standard watering regime of 12 minutes, twice daily. There were five replicate plants/time/treatment. Four separate experiments were carried out (two each in 2020 and 2021). Treatments were applied to *Actinidia chinensis* var. *chinensis* 'Zesy002' plants over 96 h **(A)** and over 112 h **(B)** in 2020. An identical experimental setup was used to compare responses between 'Zesy002' **(C)** and *Actinidia chinensis* var. *deliciosa* 'Hayward' **(D)** in 2021. Different lettering over the bars indicates statistically different differences, as shown by LSD, $p \leq 0.05$, within each experiment.

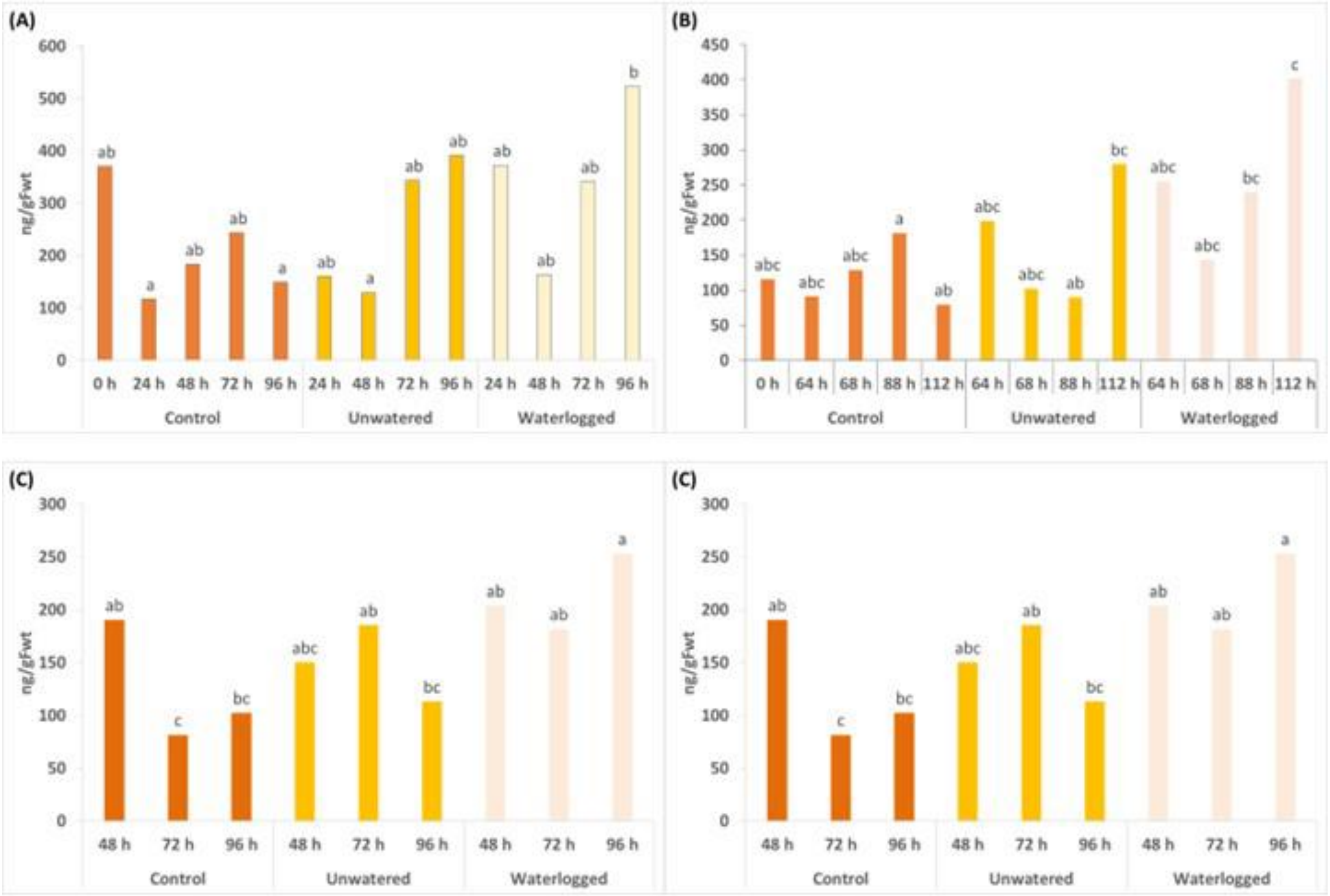

Figure S5. Salicylic acid O-β-glucoside (SAG) concentrations measured in roots of potted kiwifruit plants at various sample times (shown in hours on the x-axes) after application of three treatments. Starting from time 0 h, plants in the unwatered treatment were not watered and waterlogged plants were subjected to constant water at quarter pot height. Plants in the control treatment received the standard watering regime of 12 minutes, twice daily. There were five replicate plants/time/treatment. Four separate experiments were carried out (two each in 2020 and 2021). Treatments were applied to *Actinidia chinensis* var. *chinensis* 'Zesy002' plants over 96 h **(A)** and over 112 h **(B)** in 2020. An identical experimental setup was used to compare responses between 'Zesy002' **(C)** and *Actinidia chinensis* var. *deliciosa* 'Hayward' **(D)** in 2021. Different lettering over the bars indicates statistically different differences, as shown by LSD, $p \leq 0.05$, within each experiment.

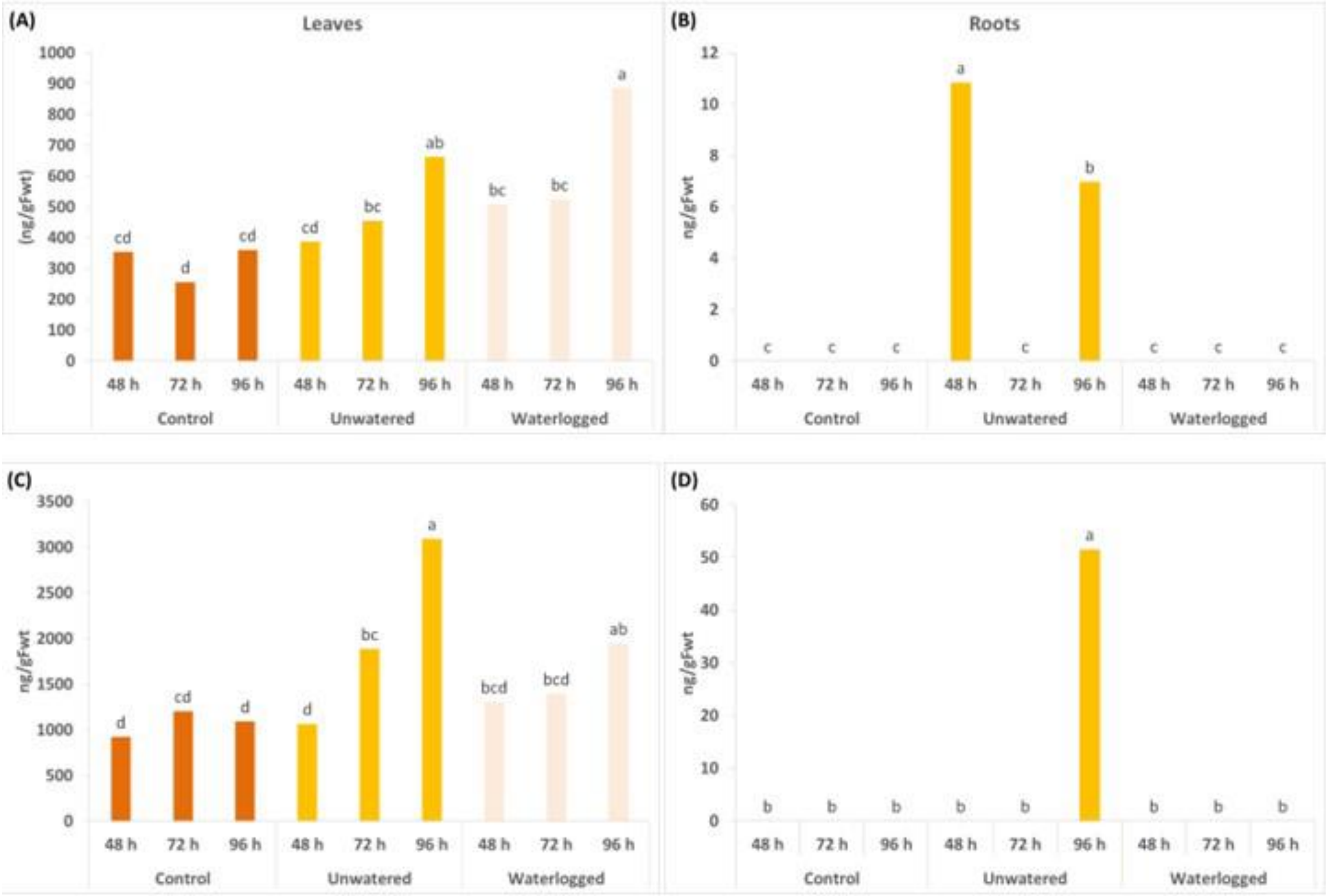

Figure S6. Abscisic acid glucosyl ester (ABA-GE) concentrations measured in leaves (left panel) and roots (right panel) of potted kiwifruit plants at various sample times (shown in hours on the x-axes) after application of three treatments in 2021. From time 0 h, plants in the unwatered treatment were not watered and waterlogged plants were subjected to constant water at quarter pot height. Plants in the control treatment received the standard watering regime of 12 minutes, twice daily. There were five replicate plants/time/treatment. Leaf and root samples came from *Actinidia chinensis* var. *chinensis* 'Zesy002', **(A)** and **(B)** respectively, and from *Actinidia chinensis* var. *deliciosa* 'Hayward' **(C)** and **(D)** respectively. Different lettering over the bars indicates statistically different differences, as shown by LSD, $p \leq 0.05$, within each tissue type and cultivar.

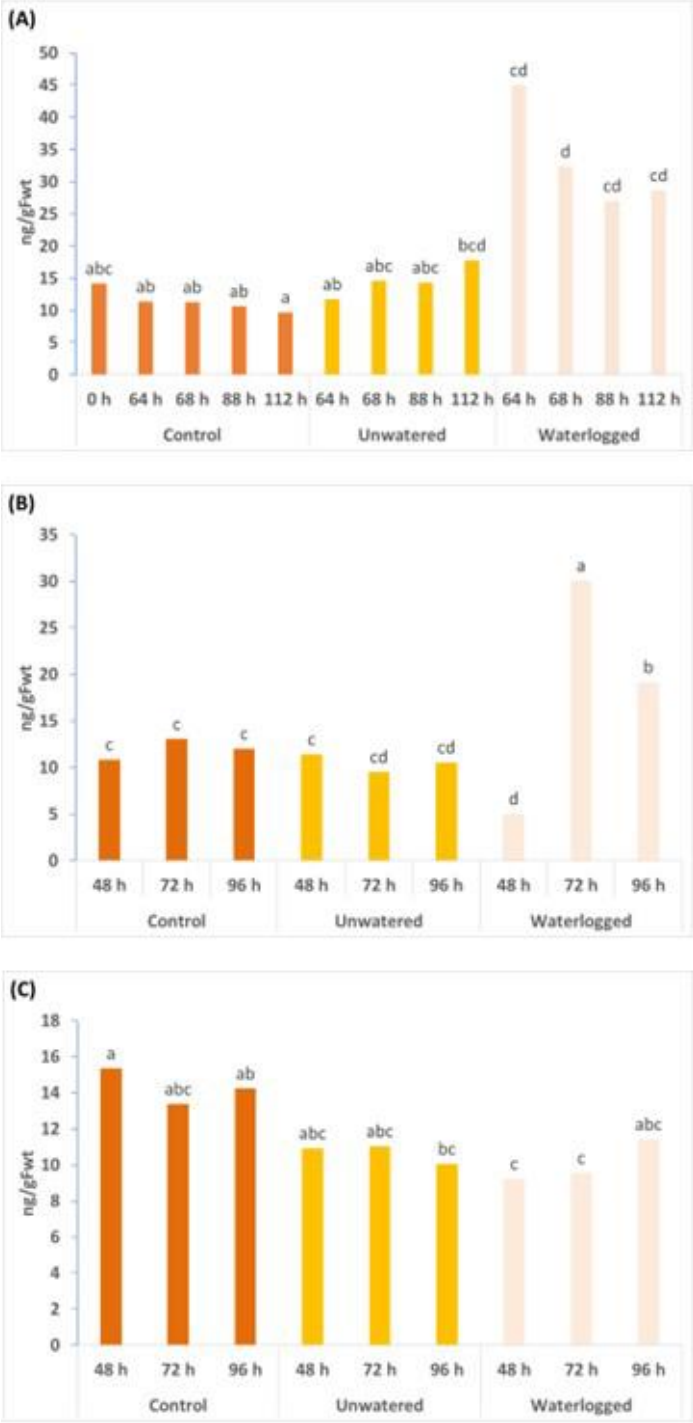

Figure S7. Indole-3-acetic acid (IAA) concentrations measured in roots of potted kiwifruit plants at various sample times (shown in hours on the x-axes) after application of three treatments. Starting from time 0 h, plants in the unwatered treatment were not watered and waterlogged plants were subjected to constant water at quarter pot height. Plants in the control treatment received the standard watering regime of 12 minutes, twice daily. There were five replicate plants/time/treatment. Results from three separate experiments are shown, with treatments applied to *Actinidia chinensis* var. *chinensis* 'Zesy002' plants over 112 h **(A)** in 2020. An identical experimental setup (treatments applied over 96 h) was used to compare responses between 'Zesy002' **(B)** and *Actinidia chinensis* var. *deliciosa* 'Hayward' **(C)** in 2021. Different lettering over the bars indicates statistically different differences, as shown by LSD, $p \leq 0.05$, within each experiment.

Table S1. *p*-Values for ANOVA tests for effects of water stress on phytohormone concentrations in the leaves and roots of *Actinidia chinensis* var. *chinensis* 'Zesy002' potted plants. Leaf and root samples were collected at 24, 48, 72 and 96 hours after water deficit or partial submergence of pots in water. Probability values lower than 0.01 are in bold.

**Experiment 1**	**Leaves**				**Roots**			
	Baseline[1]	Time	Treatment	Time x Treatment	Baseline	Time	Treatment	Time x Treatment
12-OH-JA	0.076	**0.001**	0.635	0.15	0.438	0.016	0.778	0.299
7-OH-ABA	0.044	0.156	0.057	0.136	0.528	0.049	**<.001**	0.006
ABA	**<.001**	**<.001**	**<.001**	**0.002**	**0.003**	**<.001**	**<.001**	**<.001**
ABA-GE	0.233	**<.001**	**0.001**	0.069	Below detection			
Cis-OPDA	0.284	0.060	0.331	0.300	0.575	**<.001**	0.333	0.034
DH-JA	0.818	0.204	0.609	0.554	0.021	**<.001**	**<.001**	0.054
DPA	0.408	0.052	0.061	0.291	0.387	0.070	0.041	0.052
IAA	Below detection				0.587	0.192	0.032	0.080
JA	0.004	**<.001**	0.204	0.93	0.056	0.031	**<.001**	0.480
JA-Ile	0.011	**<.001**	0.080	0.604	0.736	0.065	0.462	0.605
OPC-4	**0.003**	0.444	0.054	0.959	0.042	0.005	0.013	0.020
PA	0.940	0.629	0.696	0.031	0.589	**<.001**	**0.001**	0.115
SA	0.287	**0.009**	0.530	0.784	0.942	0.403	0.072	0.678
SAG	0.317	0.080	0.672	0.825	0.612	0.311	0.072	0.570

[1]The baseline compares the concentration of any compound to its initial concentration (at time zero)

Table S2. *p*-Values for ANOVA tests for effects of water stress on phytohormone concentrations in the leaves and roots of *Actinidia chinensis* var. *chinensis* 'Zesy002' potted plants. Leaf and root samples were collected at 64, 68, 88 and 112 hours after water deficit or partial submergence of pots in water. Probability values lower than 0.01 are in bold.

**Experiment 2**	**Leaves**				**Roots**			
	Baseline	Treatment	Time	Time x Treatment	Baseline	Treatment	Time	Time x Treatment
7-OH-ABA	0.603	**<.001**	0.417	0.421	0.342	**<.001**	0.473	**0.007**
ABA	**0.003**	**<.001**	**<.001**	0.012	0.021	**<.001**	0.019	0.507
ABA-GE	0.26	**0.005**	0.354	0.373		Below detection		
DPA	0.189	**<.001**	0.310	0.438	0.573	0.021	0.065	**0.005**
PA	0.912	0.088	**0.002**	0.089	**0.001**	**<.001**	0.642	0.428
Cis-OPDA	0.977	0.104	0.413	0.039	0.135	0.069	0.071	0.909
OPC-4	0.429	0.176	0.048	0.090	0.7	0.578	0.551	0.334
JA	**<.001**	0.388	**<.001**	0.857	0.195	**<.001**	0.469	0.58
JA-Ile	**<.001**	0.291	0.053	0.962	0.683	0.237	0.931	0.286
12-OH-JA	0.113	0.004	0.367	0.081	0.296	0.440	0.832	0.548
DH-JA	0.632	0.784	0.398	0.298	**0.006**	**<.001**	0.056	0.814
SA	**<.001**	0.884	**<.001**	**<.001**	0.25	0.044	0.194	0.097
SAG	0.956	0.019	0.341	0.9	0.987	0.025	0.549	0.53
IAA	0.290	**0.002**	**<.001**	0.505	0.677	**<.001**	0.854	0.805

[1]The baseline compares the concentration of any compound to its initial concentration (at time zero)

Table S3. *p*-Values for ANOVA tests for effects of water stress on phytohormone concentrations in the leaves and roots of *Actinidia chinensis* var. *chinensis* 'Zesy002' potted plants. Leaf and root samples were collected at 24, 48, 72 and 96 hours after water deficit or partial submergence of pots in water. Probability values lower than 0.01 are in bold.

	**Leaves**			**Roots**		
**Experiment 3**	Treatment	Time	Treatment x Time	Treatment	Time	Treatment x Time
7-OH-ABA	0.106	0.518	0.214	**0.000**	0.370	**0.000**
ABA	**0.000**	**0.000**	**0.000**	**0.000**	**0.000**	**0.000**
ABA-GE	**0.000**	**0.004**	0.341	**0.000**	**0.000**	**0.000**
DPA	**0.006**	0.209	0.564	**0.000**	0.902	**0.000**
PA	0.650	0.129	0.464	**0.000**	**0.000**	**0.000**
Cis-OPDA	0.714	0.188	0.577	0.097	**0.000**	**0.000**
OPC-4	0.231	**0.004**	0.107	**0.005**	0.272	0.220
JA	0.166	**0.000**	0.181	**0.000**	0.367	0.211
JA-Ile	0.049	**0.000**	0.091	0.192	0.626	0.591
12-OH-JA	0.723	0.013	**0.000**	**0.000**	0.022	**0.000**
DH-JA	0.718	0.556	0.258	**0.000**	**0.000**	0.047
2,5 DHBA[1]	0.042	0.047	**0.004**	0.161	0.694	0.390
SA	**0.000**	**0.008**	0.010	0.036	0.037	0.489
SAG	**0.000**	0.022	0.040	0.027	0.426	0.175
IAA	**0.000**	0.113	0.020	**0.000**	**0.000**	**0.000**

[1]Concentrations of 2,5 DHBA were often around the limit of detection

Table S4. *p*-Values for ANOVA tests for effects of water stress on phytohormone concentrations in the leaves and roots of *Actinidia chinensis* var. *deliciosa* 'Hayward'. Leaf and root samples were collected at 24, 48, 72 and 96 hours after water deficit or partial submergence of pots in water. Probability values lower than 0.01 are in bold.

	Leaves			Roots		
**Experiment 4**	Treatment	Time	Treatment x Time	Treatment	Time	Treatment x Time
7-OH-ABA	**0.000**	0.236	**0.009**	0.024	0.206	0.083
ABA	**0.000**	**0.004**	**0.003**	**0.000**	0.077	**0.000**
ABA-GE	**0.001**	**0.001**	0.079	**0.000**	**0.000**	**0.000**
DPA	**0.000**	**0.008**	**0.001**	**0.000**	0.013	**0.000**
PA	**0.000**	0.065	0.071	**0.000**	0.493	0.014
Cis-OPDA	0.111	0.660	0.131	0.052	0.093	0.980
OPC-4		Below detection		**0.000**	0.150	**0.000**
JA	0.271	0.039	0.348	**0.000**	0.085	0.037
JA-Ile	0.044	0.062	**0.007**	0.439	0.883	0.393
12-OH-JA	0.026	0.005	**0.005**	0.705	0.634	0.168
DH-JA	0.444	0.885	0.171	**0.000**	0.865	0.083
2,5 DHBA[1]	0.231	0.527	0.067	**0.007**	0.802	0.078
SA	0.020	0.445	0.434	0.254	0.415	0.112
SAG	**0.008**	0.109	0.357	**0.001**	0.149	0.012
IAA	0.544	0.585	0.968	**0.008**	0.909	0.746

[1]Concentrations of 2,5 DHBA were often around the limit of detection

Table S5. Multiple reaction monitoring (MRM) transitions used for phytohormone analysis. Optimised Q1 (precursor ion) and Q3 (product ion) transitions, retention time (RT), declustering potential (DP), entrance potential (EP), collision energy (CE) and collision cell exit potential (CXP) for each of the phytohormones analysed. The internal standard (IS) used for quantitation for each phytohormone is also given. DHBA = dihydroxybenzoic acid; SAG = salicylic acid O-β-glucoside; DPA = dihydrophaseic acid; 12-OH-JA = 12-hydroxy jasmonic acid; ABA-GE = abscisic acid glucosyl ester; PA = phaseic acid; SA = salicylic acid; IAA = indole 3-acetic acid; ABA = abscisic acid; JA = jasmonic acid; DH-JA = 9,10-dihydrojasmonic acid; JA-Ile = jasmonoyl-isoleucine; OPC-4 = (+/-)-4-(3-oxo-2-(pent-2-enyl)cyclopentyl) butanoic acid; MeJA= methyl jasmonate; cis-OPDA= cis-(+)-12-oxo-phytodienoic acid

Compound	Q1	Q3	RT (mins)	DP	EP	CE	CXP	IS
[$^2H_3$] 3,4-DHBA	156.00	112.00	1.65	-80	-10.0	-16.0	-9.0	
2,5-DHBA	153.00	109.00	2.57	-80	-10.0	-16.0	-9.0	[$^2H_3$] 3,4-DHBA
[$^2H_4$] SAG	303.00	96.90	4.53	-50	-10.0	-56.2	-8.6	
SAG	299.30	136.80	4.54	-60	-10.0	-20.0	-12.0	[$^2H_4$] SAG
DPA	281.10	237.10	4.73	-60	-5.0	-15.0	-8.0	[$^2H_6$] ABA
12-OH JA	225.10	58.90	4.78	-60	-10.0	-20.0	-7.9	[$^2H_5$] JA
ABA-GE	425.10	263.00	4.91	-60	-10.0	-15.0	-10.0	[$^2H_6$] ABA
PA	279.10	139.10	4.98	-60	-13.3	-16.2	-12.3	[$^2H_6$] ABA
[$^2H_4$] SA	141.10	97.00	4.98	-40	-7.8	-40.0	-9.6	
SA	136.90	93.00	4.99	-40	-11.0	-25.0	-7.0	[$^2H_4$] SA
[$^{13}C_6$] IAA	182.00	136.00	4.99	50	10.0	25.0	15.0	
IAA	176.00	130.00	4.99	50	10.0	25.0	15.0	[$^{13}C_6$] IAA
7-OH-ABA	279.10	151.10	5.01	-60	-5.0	-15.0	-8.0	[$^2H_6$] ABA
[$^2H_6$] ABA	269.00	159.00	5.28	-25	-10.0	-17.0	-15.0	
ABA	263.10	153.00	5.28	-60	-5.0	-15.0	-8.0	[$^2H_6$] ABA
[$^2H_5$] JA	214.00	62.10	5.51	-60	-6.0	-24.0	-8.6	[$^2H_5$] JA
JA	209.10	59.10	5.52	-60	-6.0	-24.0	-8.6	[$^2H_5$] JA
DH-JA	211.00	59.00	5.71	-100	-2.9	-20.0	-5.0	[$^2H_5$] JA
[$^2H_{10}$] JA-Ile	332.20	139.90	5.83	-60	-10.0	-26.9	-12.0	
JA-Ile	321.90	129.80	5.84	-25	-10.0	-32.0	-15.0	[$^2H_{10}$] JA-Ile
OPC-4	237.20	58.80	5.84	-25	-10.0	-23.0	-15.0	[$^2H_5$] JA
[$^2H_5$] MeJA	230.20	153.00	6.10	50	4.0	16.0	8.0	
MeJA	225.20	151.20	6.11	50	4.0	16.0	8.0	[$^2H_5$] MeJA
cis-OPDA	291.00	165.00	6.40	-48	-10.0	-26.0	-3.0	[$^2H_5$] JA

Supplementary protocol S1. Gene mining protocol for identifying genes of interest.

Methodology

Use of EnsemblPlants Compara gene trees

We identified the most likely kiwifruit orthologues of genes previously identified in the literature (mostly derived from research in the model plant species *Arabidopsis thaliana*) using the following process. Literature searches were used to identify publications that identify candidate water-stress response genes and genes involved in ABA metabolism, catabolism and regulation. The search for orthologs of these genes followed one of two potential routes. If the genes identified were from plants that were not present within the fully sequenced genomes lodged in the Ensembl Plants database (htpps://plants.ensembl.org) we used BlastP to identify the most likely candidate genes from the same species or a close relative that was present in this database. If the genes identified were from plants that were present within the fully sequenced genomes lodged in Ensemble, then we searched for these genes themselves in the Ensembl Plants database.

The phylogenetic trees already constructed from these gene sequences present in Ensembl Plants under the Plant Compara (Gene Tree) displays were utilized to find the likely nearest relatives of this gene in the Asterid Kiwifruit genome represented in Ensembl Plants (*Actinidia chinensis* var *chinensis* Red5 PS1 1.69.0). First, the query gene itself and other branches of genes containing its closest relatives in the same species of origin were identified in these tree displays. We then identified the nearest branch in this tree that contained Asterid species (labelled as Asterid or with a less specific label that indicated it might contain Asterids e.g., Pentapetalae) and checked that it included potential kiwifruit orthologs). We noted if this branch was closer than the next nearest branch containing relatives from the same species of origin as the query gene. The next nearest branch containing a different clade of kiwifruit genes was also identified (if one was present in the same tree). If this kiwifruit branch was close enough to potentially include an orthologue of the query gene, then it was also included. If it was clearly separated with several basal plant species grouping near the root of the tree with the first kiwifruit clade it was not included. The tree was then examined to see if it was consistent with the phylogenetic expectations based on the relationships of the plant species within the tree and to identify and take into account likely duplication events/gene radiation events within the species from which the query sequence was derived and any other plant species. The likely impact of any such amplifications on finding the nearest kiwifruit neighbor was then taken into account in the subsequent analysis. The structure of the tree was also examined to see if it was generally consistent with the expected evolutionary relationships between the clades of genes based on knowledge of the phylogenetic relationships between the plant species in EnsemblPlants. In most cases these expectations were met after examining the branches containing the primary query sequence. In a few cases more cent duplication events present in the species of origin of the query sequence but not in more distantly related species meant that neighboring clades contained the most likely kiwifruit orthologues. This scenario was quite common for *Arabidopsis*-derived query sequences which regularly show this type of duplication/amplification when compared to more distantly related Rosid and Asterid plant species.

Gene synteny analysis using Genomicus Plants

Gene pairs were also examined in Genomicus Plants v49.01 (https://www.genomicus.bio.ens.psl.eu/genomicus- plants-49.01). If present in this database. the original query sequence was first used as the reference gene in this database. If not present the nearest neighbor present in this database was used (as described for the EnsemblPlants database). We looked for evidence of micro-synteny between the source species and kiwifruit around the query gene itself. If none was present, the next-door neighboring genes were also examined to see if evidence traces of synteny in the region could be found. Gene radiation events in either species as well as other Asterid and/or Rosid species were also obvious from these comparisons and were thus noted as likely to affect the ability to identify the loci likely to be orthologous by descent as well function. This evidence was combined with evidence from the EnsemblPlants trees. Genomicus Plants also allowed us to easily determine if they are likely descended from a whole genome *Actinidia* genome duplication event that is present early in the evolution of this species and results in homoeologous genome regions on at least two chromosomes. In such cases a significant degree of synteny could be detected on both sides of the *Actinidia* gene when selecting one of the *Actinidia* genes as the reference gene.

Genes chosen for inclusion in the NanoString array

The NanoString array used for this analysis had a limited number of positions available for candidate genes. The candidate genes chosen therefore had to prioritized according to a number of factors. It was not possible to include a large number of candidate genes for any particular kiwifruit gene family. The strategy was therefore to include genes with a smaller number of potential candidate genes and exclude those with a large number of potential candidates to fit the Nanostring design of 18 GoIs. The genes chosen for inclusion in the NanoString array were then selected based on a combination of the strength of the data in the model species identifying those genes, the presence of any micro-synteny, and the likelihood that the orthologue could be identified from the phylogenetic and comparative genome analyses performed. Micro-synteny in the neighboring gene region can be considered as independent evidence that does not rely on the tree alignment data for the query gene. From this data, the sequence(s) most similar by alignment was (were) chosen as the most likely candidate(s) of the query gene and incorporated into the NanoString gene-sets. Sometimes multiple kiwifruit genes equally close were added to the gene set. Table 3 lists the genes used in the NanoString gene-sets. EnsemblPlants trees and Genomicus plants alignments are shown in the below sections detailing the analyses for individual genes. The genes analysed in this section include all 12 of the kiwifruit genes identified in this analysis for the first time (included in Table 3 under the column "reference for kiwifruit genes identified. It also includes a subset of three kiwifruit ABA/drought stress genes which have been published previously. These were checked with the same analysis to make sure that they identified one of the most likely functional orthologues of the original gene (typically from *Arabidopsis*) where the function was first ascribed.

NCED3 EnsemblPlants Plant Compara Tree and GenomicusPlants comparisons.

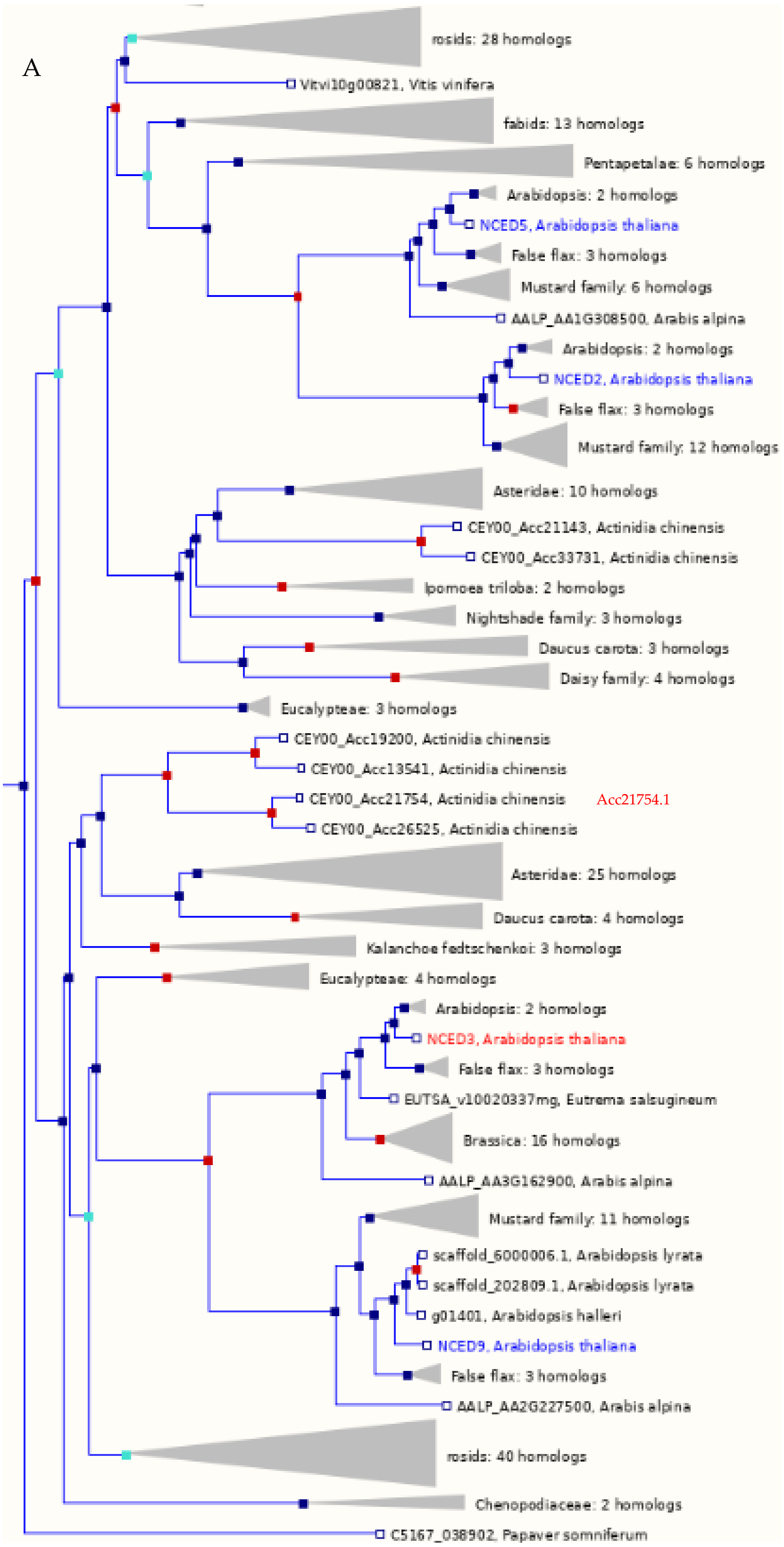

LEGEND: A) A subsection of Plant Compara Gene Tree EPIGT00950000210233 identifying the query gene (*A. thaliana NCED3* in red) its closest *Arabidopsis* neighbor (*NCED9* in blue below *NCED3*), the nearest clade of kiwifruit orthologues to *NCED3* and *NCED9* (Acc19200, Acc13541, Acc21754 and Acc26525) as well as additional clades of more distant *Arabidopsis* clades (*NCED5* and *NCED2* in blue above *NCED3*) and an additional kiwifruit clade (Acc21143, Acc33731) that is more likely to be orthologous to *Arabidopsis NCED5* and *NCED2*. Other clades identified on the tree were also searched for kiwifruit or *Arabidopsis* members and are collapsed for clarity where they contained member of neither of these two species. A gene from a basal dicot plant species (*Papaver somniferum*) lies at the base of this tree of other dicot members and so that fits with this being ancestral to all of these genes from a phylogenetic perspective. B) The Genomicus Plants v49.01 analysis shows a degree of micro-synteny in kiwifruit with the original locus (*AtNCED3* in this case), making it more likely that the matching set of kiwifruit genes have a common ancestry with the query gene and are related by descent. Six additional loci in kiwifruit show micro-syteny in the region surrounding the kiwifruit *NCED3* candidates. When aligned within kiwifruit, the *AcNCED3* candidates Acc19200 and Acc13541 form a gene pair present at homoeologous genome locations in kiwifruit showing large regions of synteny on Linkage Groups (LG) 17 and 12 respectively, while Acc21754 and Acc26525 form another gene pair present at homoeologous genome locations in kiwifruit on LGs 29 and 23 respectively. In each case the lower gene was aligned by selecting the top gene in Genomicus.

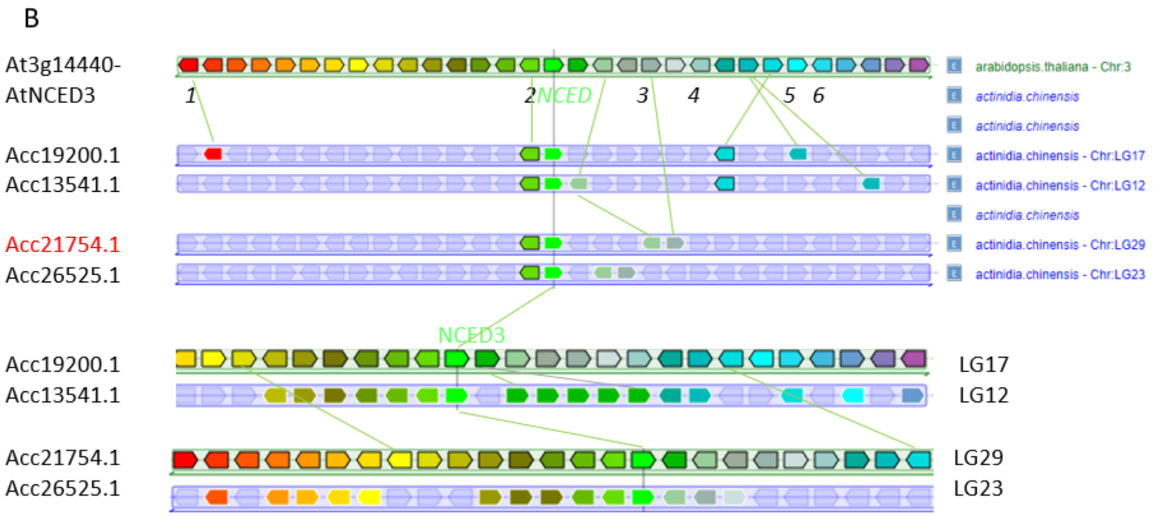

The NCED3 *Arabidopsis* clade contains its duplicated paralogue NCED9 in *Arabidopsis* and shows this is a Brassicaceae specific duplication. The three clades of plant genes that group next to the likely kiwifruit NCED3/9 orthologues in the Ericales basal asterid species of kiwifruit (namely from the basal campanulid asterid *Daucus carota*, the basal Eurosid Saxifragales species *Kalanchoe fedtschenkoi* and a clade containing the remaining Asteridae) fit from the phylogenetic perspective of the likely distant evolutionary relationships between these sets of species. Furthermore, the relationships of the other clades within the Rosids nearer to the Arabidopsis NCED3 and NCED9 clades also fit from the phylogenetic perspective of the likely evolutionary relationships between these sets of species. This indicates that the tree is likely to be a good representation of the deduced evolutionary relationships between these genes. The kiwifruit candidate Acc21754.1 *AcNCED3* was selected as the NanoString candidate.

ABA1-ZEP EnsemblPlants Plant Compara Tree and GenomicusPlants comparisons.

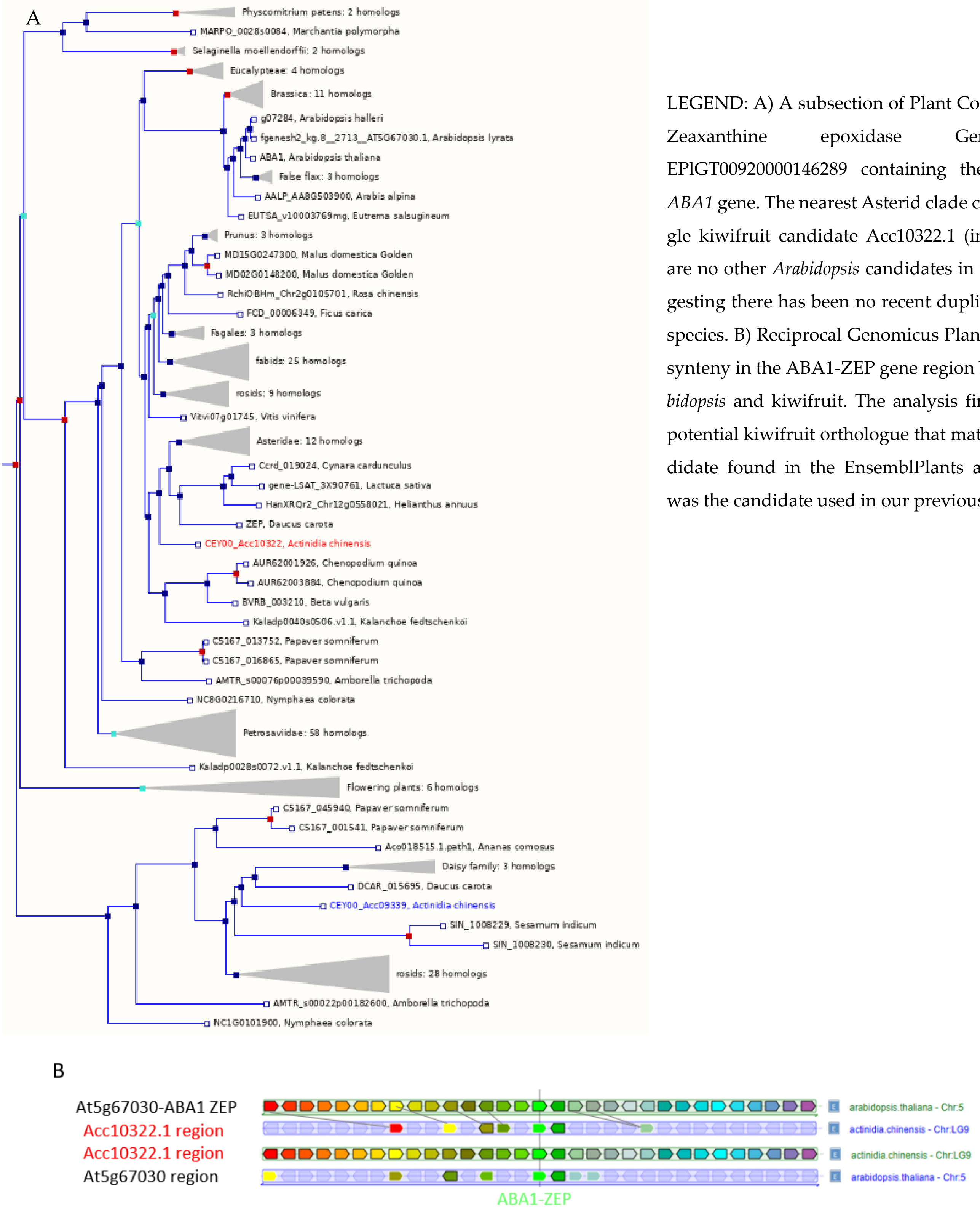

LEGEND: A) A subsection of Plant Compara ABA1 Zeaxanthine epoxidase Gene Tree EPlGT00920000146289 containing the *Arabidopsis ABA1* gene. The nearest Asterid clade contains a single kiwifruit candidate Acc10322.1 (in red). There are no other *Arabidopsis* candidates in this tree suggesting there has been no recent duplication in this species. B) Reciprocal Genomicus Plants analysis of synteny in the ABA1-ZEP gene region between *Arabidopsis* and kiwifruit. The analysis finds only one potential kiwifruit orthologue that matches the candidate found in the EnsemblPlants analysis. This was the candidate used in our previous analysis [1].

ABF4 EnsemblPlants Plant Compara Tree and GenomicusPlants comparisons.

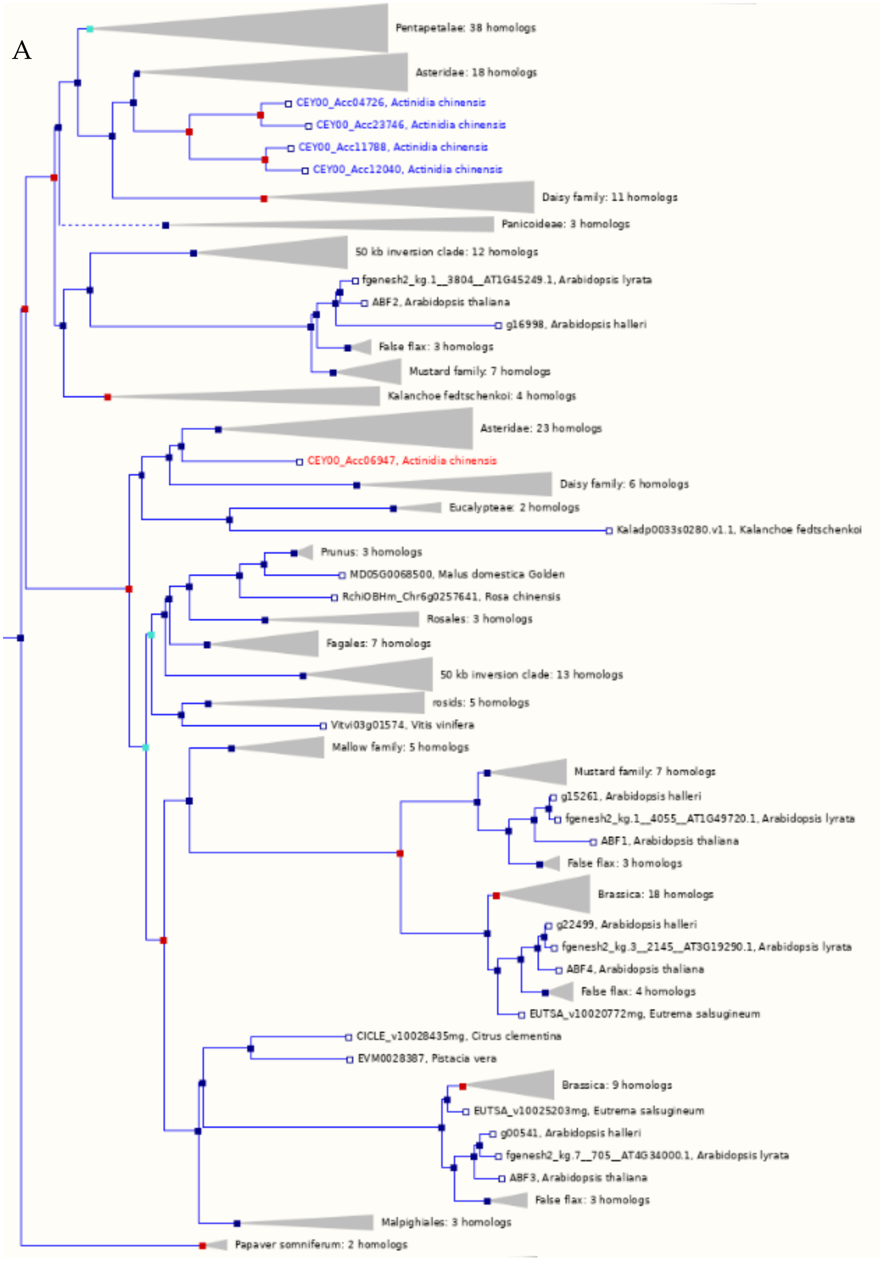

LEGEND: A) A subsection of Plant Compara ABF4 tree EPlGT00940000164340. The ABF4 tree contains 3 closely related sequences in *Arabidopsis* (*ABF1*, *ABF3* and *ABF4*) that are the result of a recent amplification in Brassicaceae. A fourth gene groups separately and is present in other species as well. The kiwifruit candidate Acc06947.1 is the only likely orthologue based on this tree. B) As expected *ABF1* and *ABF4* showed no syntenic regions in kiwifruit, while *ABF2* showed micro-synteny with the four kiwifruit genes that group with this gene in EnsemblPlants (not shown). The most likely kiwifruit candidate identified on the tree shows micro-synteny with only one of the three *ABF* candidates from *Arabidopsis* (*ABF3*). It is likely that the other two loci in *Arabidopsis* have moved genome location during the amplification process. A Rosid species with just one *ABF1/3/4* candidate (*Malus domestica*) also shows micro-synteny with the same kiwifruit gene, making it more likely that this is the orthologue by descent of all three *Arabidopsis* genes including the query gene *ABF4*.

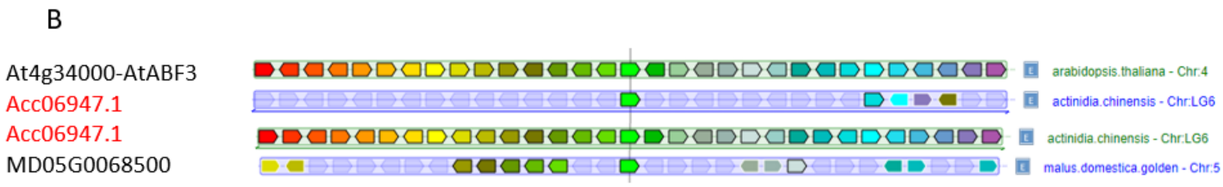

DREB2 EnsemblPlants Plant Compara Tree and GenomicusPlants comparisons.

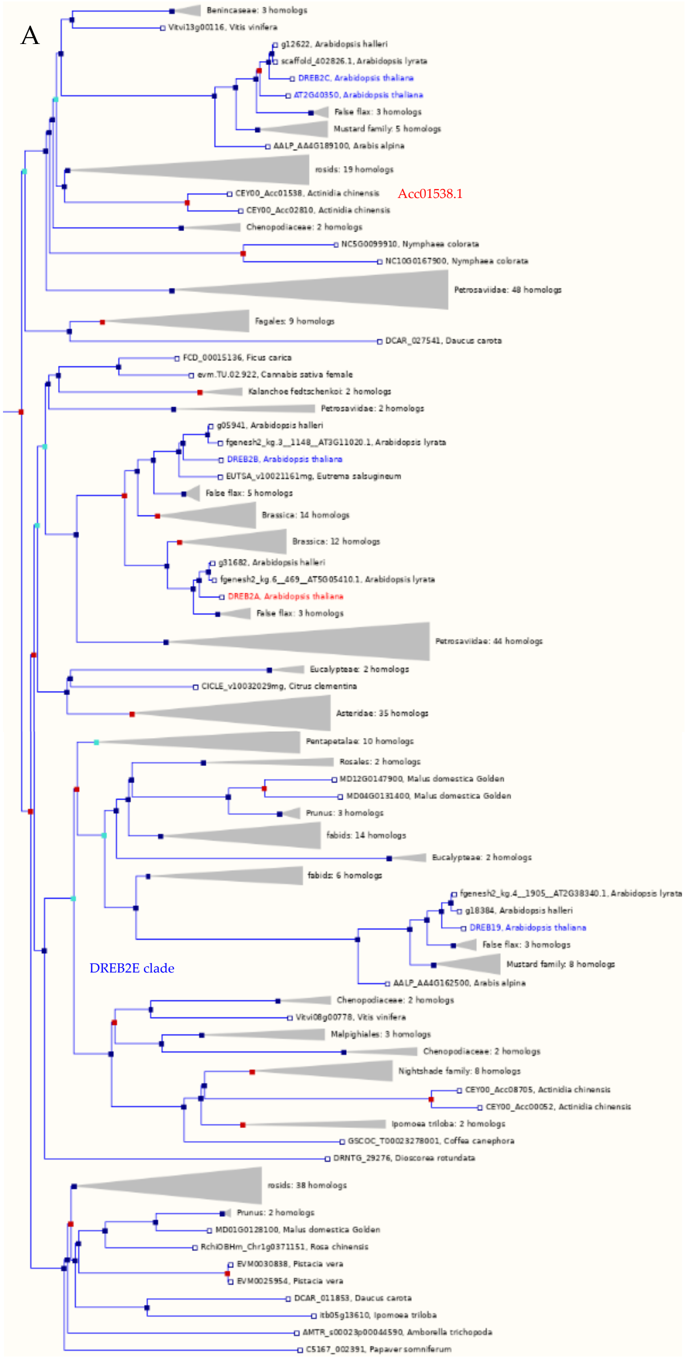

LEGEND. A) A subsection of Plant Compara DREB2 tree EPlGT00950000210497. The original query gene for this analysis was the *Arabidopsis DREB2A* gene since *DREB2A* and *DREB2B* are well known as drought responding genes in *Arabidopsis*. The DREB2A and DREB2B clades are largely restricted to the Brassicaceae. The next closest clade is the DREB2E clade followed by the DREB2C clade. The *DREB2C* gene in *Arabidopsis* also has links to ABA and other stresses. It binds to *bZIP ABF2* and together they control ABA sensitivity. *DREB2C* overexpression lines are also altered in other stress response and the plants are dehydration sensitive while *DREB2C* also binds to *ABF3* and *ABF4* [2]. The DREB2E clade has two kiwifruit candidates Acc00052 and Acc08705. The DREB2C clade has two kiwifruit candidates Acc01538 and Acc02810. B) The Genomicus synteny analysis also suggests *DREB2A* and *DREB2B* are restricted to Brassicaceae and *DREB2C* does not find any *Actinidia* members either. As the DREB2C and DREB2E clades are still quite closely related by descent we also searched for micro-synteny there. There are small levels of micro-synteny detectable between Rosid DREB2E (e.g., from apple) and the two sets of kiwifruit candidate genes. There is also micro-synteny between the "gene neighborhood" of DREB2C and the neighbor of Acc01538.1 (Acc01537.1). This suggests this gene family has been derived by multiple duplications and shows substantial gene loss in selected plant lineages giving rise to punctuated gain and loss signals that have probably led to this pattern. Note, although the original gene used as the query was *DREB2A* since there is no formal orthologue to *DREB2A* outside of the Brassicaceae we have referred to this using the more general name of "*DREB2*". The selected gene (Acc01538.1) was chosen by alignment similarity.

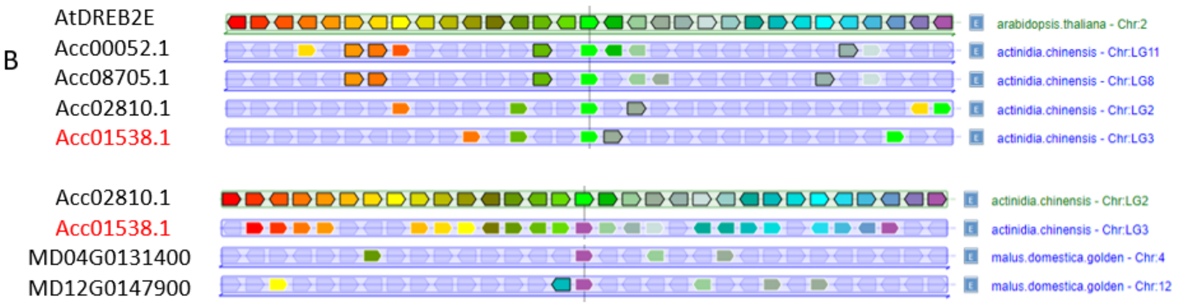

RD22 EnsemblPlants Plant Compara Tree and GenomicusPlants comparisons.

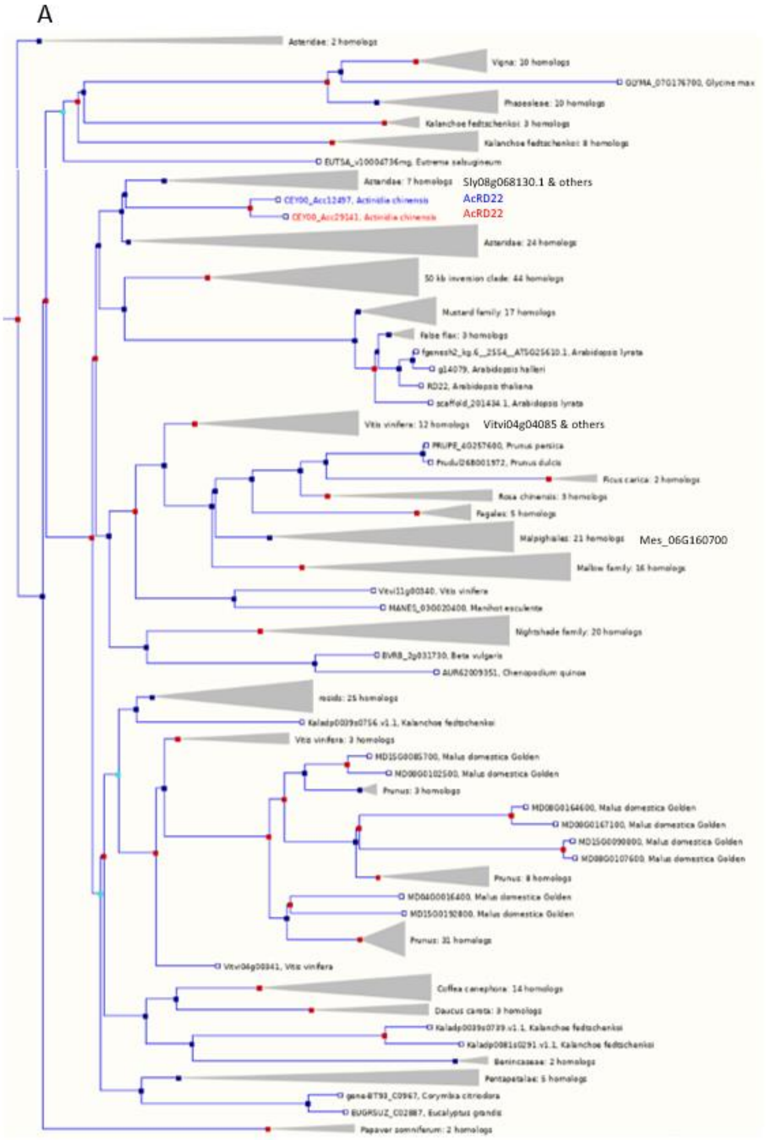

LEGEND A) Subsection of GeneTree EPlGT00950000210332 containing the BNM2, USP, RD22, and PG1beta (BURP) domain containing RD22 tree. It contains basal species like *Papaver somniferum* (basal dicot) and *Kalanchoe fedtschenko* (basal Saxifragales) at the base of the tree and shows significant additional radiation in some species. The next nearest branch of this tree (not shown) contains another BURP domain protein family USP/USPL1 which shows an even more extreme radiation pattern and contains two other kiwifruit members Acc07338 and Acc07026 that clearly group outside of the RD22 tree. B) Genomicus plant analysis showing only a small degree of micro-synteny between different species but rapid evolution and radiation of RD22 (probably by multiple duplication events and probably re-arrangements) in many species and explaining why its ancestry is more difficult to trace. There is also more evidence of micro-synteny between the region containing the *AcRD22* candidates and *Arabidopsis RD22* if you look beyond the region immediately adjacent to *RD22* and this is re-enforced by further micro-synteny in *Vitis* chromosome 4 as well beyond the region where the *RD22* gene has amplified by a series of tandem duplications. Acc29141.1 was selected as the NanoString candidate and this was the same candidate used in our previous analysis [1].

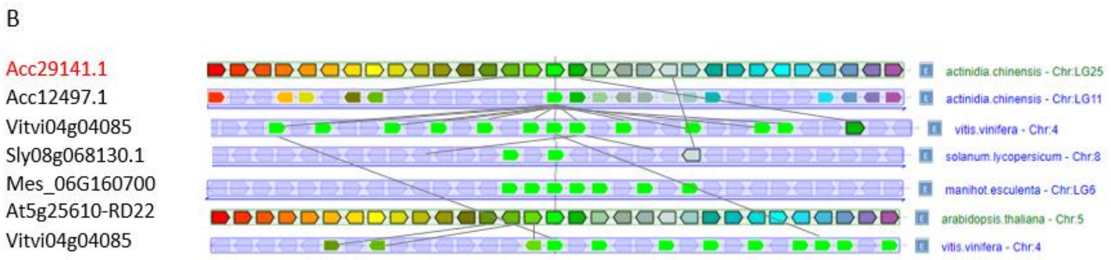

RD29B/LT165 EnsemblPlants Plant Compara Tree and GenomicusPlants comparisons.

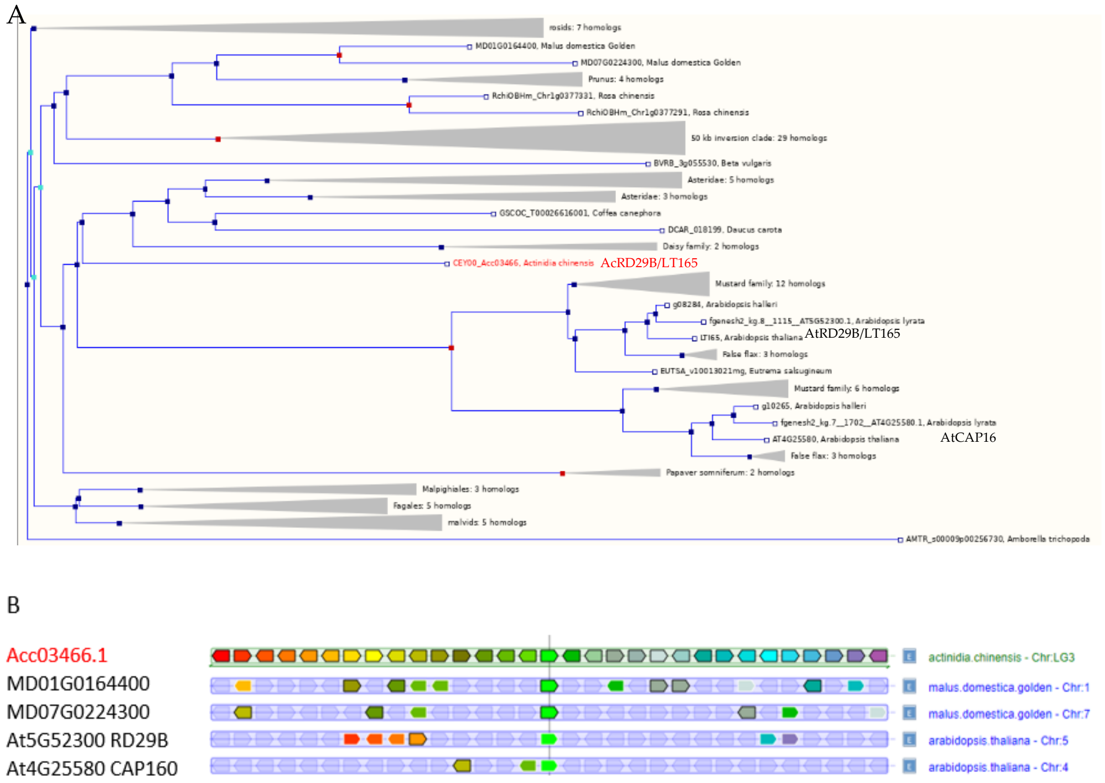

LEGEND A) Complete Plants Ensembl GeneTree EPlGT00140000004826 containing RD29B/LTI65 and the related AtCAP160 (homologous to the spinach CAP160 drought induced gene both RD29B and CAP160 contain the Low-Temperature Induced or LTI domain). This tree contains just a single orthologue from kiwifruit. B) Genomicus plant analysis of Acc03466.1 showing reasonable levels of micro-synteny with Rosid plants and with the RD29B and CAP160 drought induced loci in particular This clearly shows that this kiwifruit locus is related by descent to (and the orthologue of) the RD29B/CAP160 family. Since there was only a single candidate, Acc03466.1 was selected as the NanoString candidate.

WRKY40 EnsemblPlants Plant Compara Tree and GenomicusPlants comparisons.

A

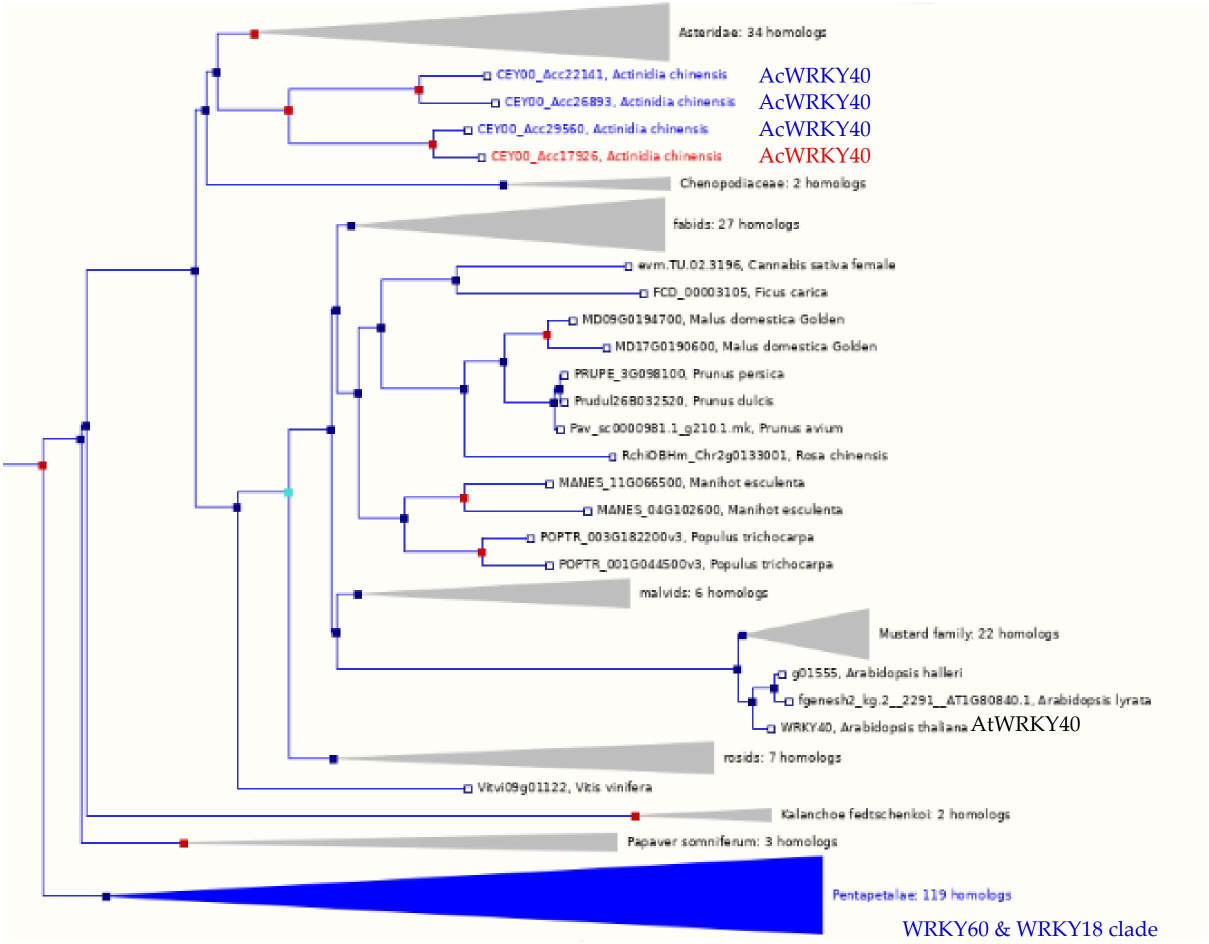

B

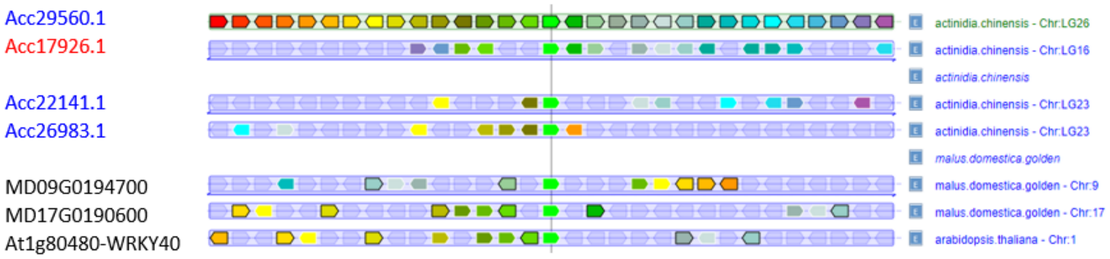

A) Subsection of the GeneTree EPlGT00940000164102 containing the WRKY40 family of transcription factors. It contains basal species like *Papaver somniferum* (basal dicot) and *Kalanchoe fedtschenko* (basal Saxifragales) at the base of the tree and a large outgroup that contains the related WRKY18/60 family. B) Genomicus plants analysis showing reasonable micro-synteny can be detected between the four kiwifruit (Asterid) candidates and Rosid candidates including the query gene *AtWRKY40* showing that these four kiwifruit loci are related by descent to (and the orthologues of) the AtWRKY40 family. Acc17926.1 was selected as the NanoString candidate.

CYP707A EnsemblPlants Plant Compara Tree and GenomicusPlants comparisons

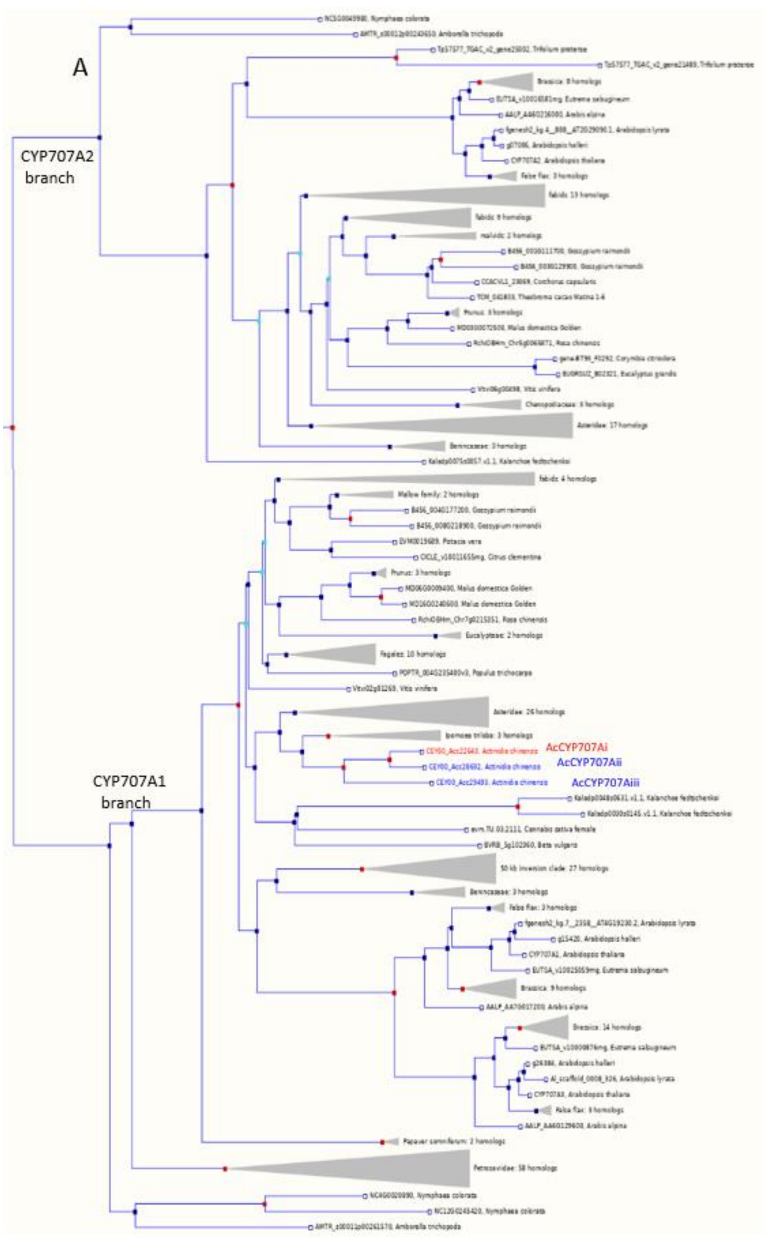

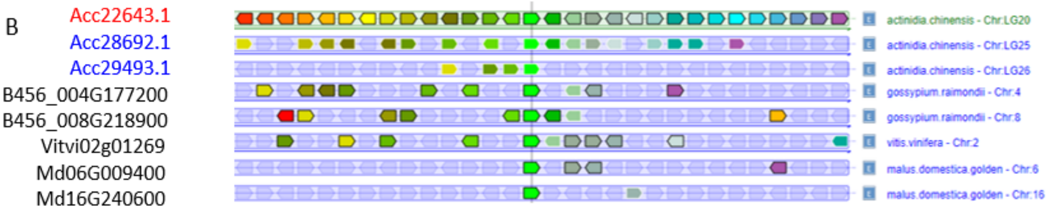

LEGEND A) Subsection of the Gene Tree EPlGT00940000164464 containing the CYP707A1 and A2 branches of P450 genes. The A2 branch does not contain any kiwifruit members and the A1 branch has one clade of closely related kiwifruit candidates embedded within the CYP707A1 Asterid sub-branch of this tree as would be expected. Their position within this sub-branch is consistent with the known phylogenetic relationships between the taxa in that clade. B) Genomicus plants analysis showing reasonable micro-synteny can be detected between the three kiwifruit (Asterid) candidates and other Rosid candidates from the CYP707A1 sub-branch. However there is no micro-synteny with the two *Arabidopsis* branch members CYP707A1 and CYP707A3. The Brassicaceae members of this subclade do not show any synteny outside of the Brassicaceae, indicating that the ancestral gene that gave rise to this clade in the Brassicaceae was likely moved to a novel genome location before this clade evolved further. All three kiwifruit candidates showed significant micro-synteny between each other with the two candidates closest to each other (other Acc22643.1 and Acc28692.1) showing the most extensive micro-synteny and probably derived from the most recent *Actinidia* genome wide duplication event. Acc22643.1, Acc28692.1 and Acc29493.1 were all selected for the NanoString analysis.

ABI4 EnsemblPlants Plant Compara Tree and GenomicusPlants comparisons.

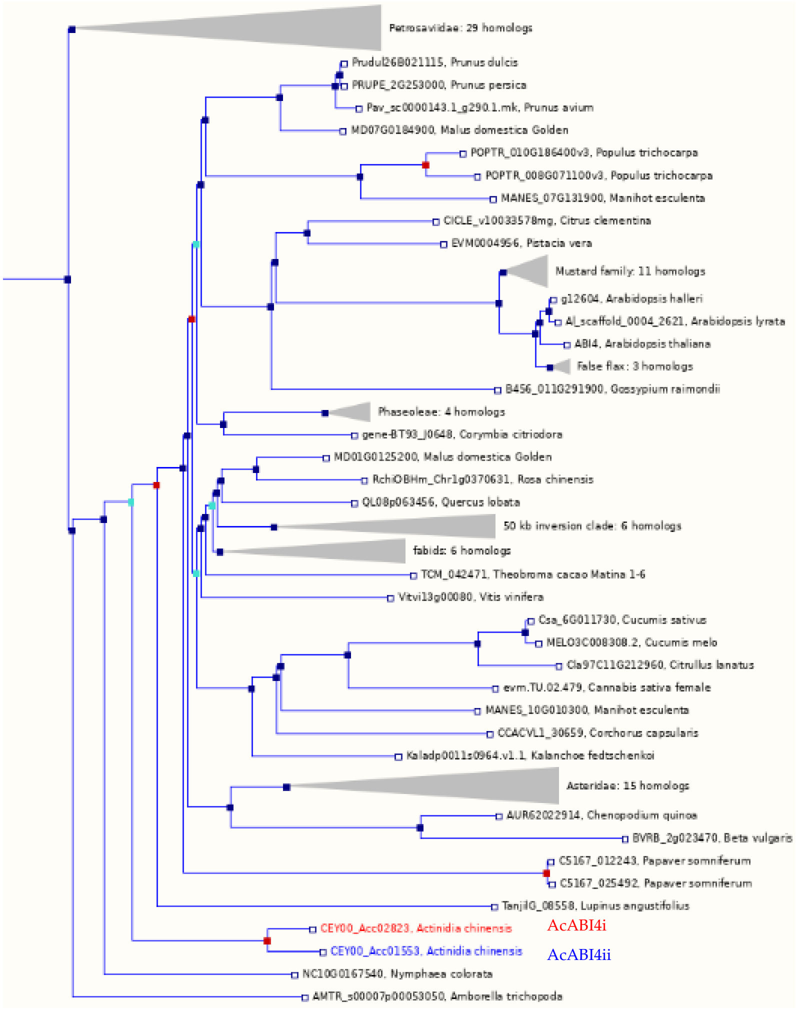

LEGEND. A) A second subsection of Plant Compara DREB2 tree EPlGT00950000210497. ABI4 is another branch of the DREB2 tree (see above). There are only two kiwifruit candidates on this branch of the tree. They group near the Asterid clade of ABI4 candidates, albeit in a more basal position than expected from the known phylogenetic relationships between the taxa. As they are the only candidates that makes it likely they are the orthologues of ABI4. B) Genomicus plants analysis showing reasonable micro-synteny can be detected between the two kiwifruit (Asterid) candidates, the *Arabidopsis* gene used as the query (*ABI4*) and other Rosid candidates from the ABI4 branch. Given this micro-synteny it is likely that both kiwifruit candidates (Acc02823.1 and Acc01553.1) could function as orthologues of ABI4. They were both chosen for NanoString analysis.

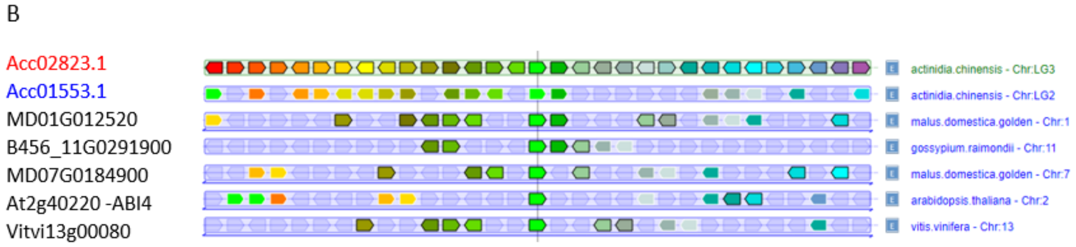

UTC-71 EnsemblPlants Plant Compara Tree and GenomicusPlants comparisons.

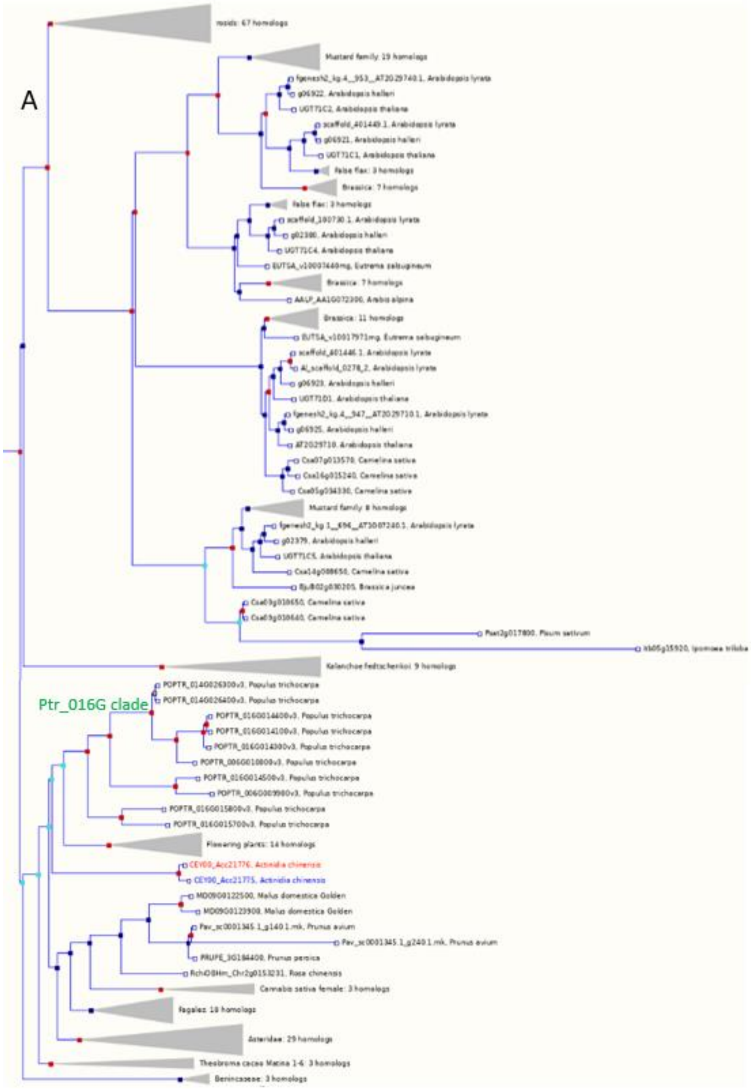

LEGEND. A) A subsection of Plant Compara tree number EPlGT00950000210342 that belongs to a very large super-tree of more than 23,000 plant genes. Like other UTC-71 families, it is therefore no surprise that the UTC-71C family appears to have diverged very substantially. Commensurate with this, obvious orthologues by either genome position (see below) or tree position are difficult to find. Most of the duplication in this family seems to be specific to Brassicales or close relatives of the Brassicales like the Malvales. B) Genomicus plants analysis showing substantial amplification can be seen in plant species such as Poplar (LG16). The cases of gene radiation, re-arrangements and evidence of selection in these genes probably makes it quite possible that the function of this clade may have been transferred to a neighboring clade. The kiwifruit candidate we tested (Acc21776.1) was the closest to the *Arabidopsis UTC-71C5* candidate by its tree position (with another candidate right next to it equally close). The other most likely alternative gene models that may carry out the ABA glycosylation function are discussed in the section below. Note that this family is also identified as UGT71K1 in some species.

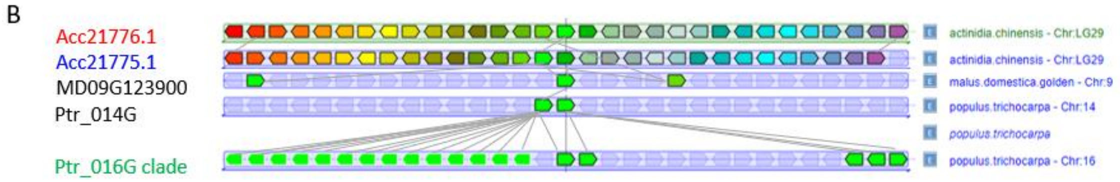

Other potential candidates that may glycosylate ABA.

One of the genes in the clade with Acc26543.1, Acc26544.1, Acc26545.1 and Acc26546.1 which group with the UTC-71B/HYR family are the second most likely to perform the ABA -glycosylation role in other species like kiwifruit. This clade contains a gene (*AtUTC-71B6*, LG3) that has already been shown to be able to glycosylate the natural (+) enantiomer of ABA. Unfortunately, there is no convincing orthologue of the UTC-71B6 candidate in kiwifruit either with members showing equally rapid radiation and evolution to the UTC-71C family. The above kiwifruit orthologues however do match with regions of *Arabidopsis* with many "missing" orthologous gene models in an *A. thaliana* genome region that contains *UTC-71B* candidates (LG3 between At3G17730 and At3G22170). These regions of micro-synteny with other neighboring genes are in the same regions as many of the *UTC-71B* models (At3g21750- At3g21800). This suggests there may actually be a relationship by descent that has eroded the most obvious signs of their close relationship on the phylogenetic tree and matches with expectation given the multiple signatures of rapid gene radiation discussed above.

RBOH-F EnsemblPlants Plant Compara Tree and GenomicusPlants comparisons.

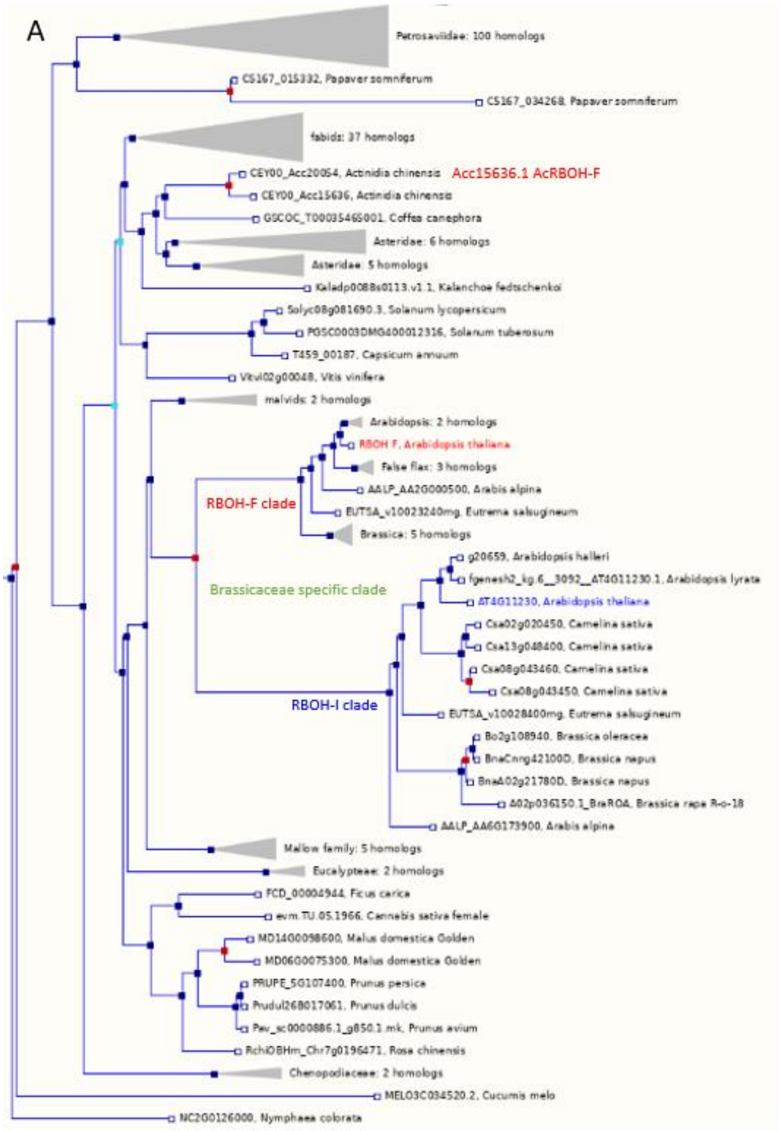

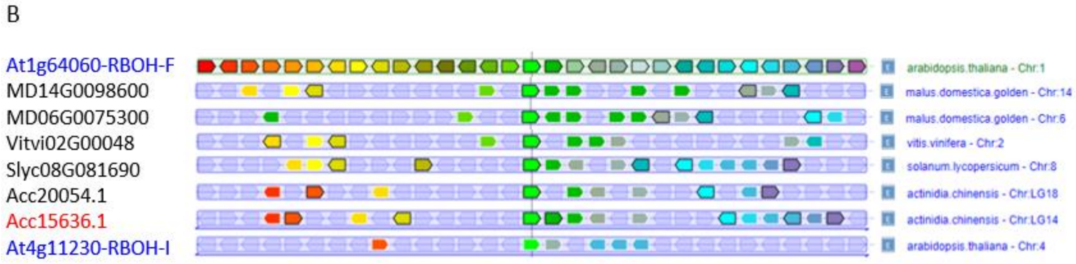

LEGEND A) Partial Plant Compara GeneTree EPlGT00940000163337 containing the RBOH family including RBOH-F and RBOH-I. The duplication of RBOH-F and RBOH-I is a Brassicaceae specific event and therefore non-Brassicaceae are likely to be equally close to either of these two genes. There are only two kiwifruit candidates on this branch of the tree, Acc20054.1 and Acc15636.1. They group within the Asterid clade of RBOH-F and RBOH-I candidates as expected from the with the known phylogenetic relationships between the taxa. This makes these two genes the likely orthologues of RBOH-F in kiwifruit. B) Genomicus plants analysis shows significant retention of micro-synteny between the Arabidopsis RBOH-F locus, other Rosids, as well as both kiwifruit candidates. These candidates actually show a greater level of micro-synteny than RBOH-F shows with its sister locus in Arabidopsis RBOH-I. This is consistent with these two kiwifruit loci being descendent from the same ancestral gene locus as RBOH-F/I. Acc15636.1 was selected as the NanoString candidate and this was the same candidate used in our previous analysis [3].

ETR1 EnsemblPlants Plant Compara Tree and GenomicusPlants comparisons.

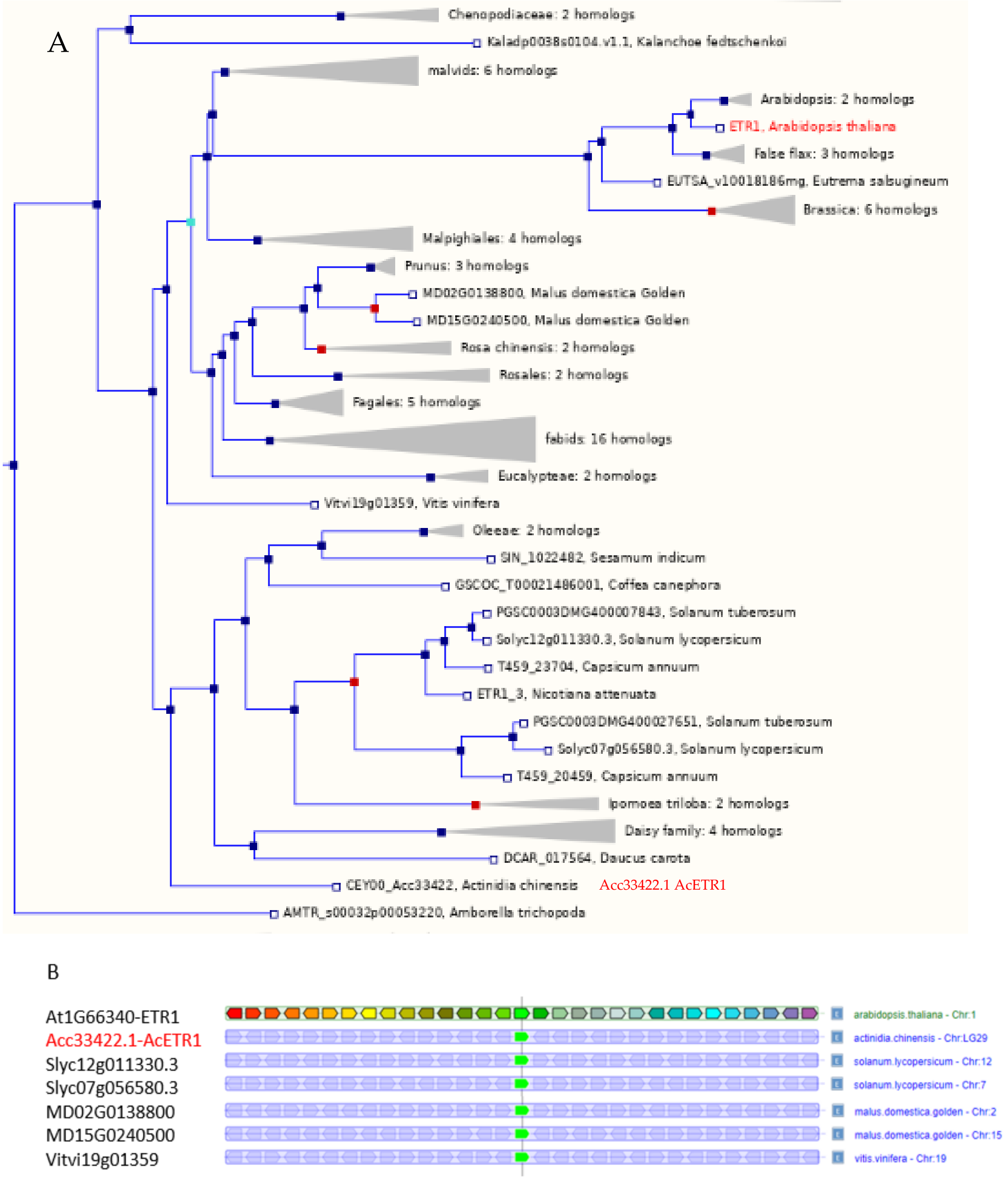

LEGEND A) Partial Plant Compara GeneTree EPlGT00940000165484 containing the Ethylene receptors ETR1 and its relative ERS1 (this part of the tree is not shown). There is only one kiwifruit candidate on this branch of the tree, Acc34422.1. It groups basally to most of the Asterid clade of *ETR1* candidates as expected from the known phylogenetic relationships between the taxa. This makes this gene the likely orthologues of *ETR1* in kiwifruit. Notably this branch does not contain any monocots. The component of the tree carrying the ERS1 orthologues was also checked and this is clearly separated from the ETR1 tree and contains basal plant species including a basal dicot and a large monocot clade. B) The Genomicus plants analysis shows no micro-synteny between the *Arabidopsis ETR1* locus and any of the species outside of the Brassicaceae. The synteny data therefore does not add any extra evidence in this case. Similarly, the analysis of the ERS1 genome Region does not show any extensive micro-synteny across plant families. Since there was only a single *ETR1* candidate, Acc33422.1 was selected as the NanoString candidate.

Jasmonate signaling pathway

Note the gene we have used to assess signaling by the jasmonate pathway is not the MYC2 gene often used in *Arabidopsis* but a sister gene from the MYC2-like clade that is also known as bHLH14. This gene was identified by a comprehensive analysis of candidate genes [4] and will be referred to as MYC2-like.

References specific to the Supplementary protocol

1. Wurms, K.V.; Hardaker, A.J.; Ah Chee, A.; Bowen, J.; Phipps, J.; Taylor, J.; Jensen, D.; Cooney, J.; Wohlers, M.; Reglinski, T. Phytohormone and putative defense gene expression differentiates the response of 'Hayward' kiwifruit to Psa and Pfm infections. *Front. Plant Sci.* **2017**, *8*.
2. Lee, S.J.; Kang, J.Y.; Park, H.J.; Kim, M.D.; Bae, M.S.; Choi, H.I.; Kim, S.Y. DREB2C Interacts with ABF2, a bZIP Protein Regulating Abscisic Acid-Responsive Gene Expression, and Its Overexpression Affects Abscisic Acid Sensitivity. *Plant Physiol.* **2010**, *153*, 716-727, doi:10.1104/pp.110.154617.
3. de Jong, H.; Reglinksi, T.; Elmer, P.A.G.; Wurms, K.; Vanneste, J.L.; Guo, L.F.; Alavi, M. Integrated use of *Aureobasidium pullulans* strain CG163 and acibenzolar-S-methyl for management of bacterial canker in kiwifruit. *Plants* **2019**, *8*, doi:10.3390/plants8080287.
4. Stroud, E.A. Understanding the delicate balance between microbial pathogens and insect pests and optimising protection in *Actinidia chinensis*. University of Auckland, 2023.
